# Randomizing the growth of silica nanofibers for whiteness

**DOI:** 10.1016/j.xcrp.2024.102021

**Published:** 2024-06-19

**Authors:** Zhen Lin, Johannes S. Haataja, Xichen Hu, Xiaodan Hong, Olli Ikkala, Bo Peng

**Affiliations:** 1 Department of Applied Physics, Aalto University, P.O. Box 15100, 02150 Espoo, Finland; 2 Department of Materials Science, Advanced Coatings Research Center of Ministry of Education of China, Fudan University, Shanghai 200433, China

**Keywords:** colloids, light scattering, random curliness, silica nanofibers, whiteness

## Abstract

In colloids, the shape influences the function. In silica, straight nanorods have already been synthesized from water-in-oil emulsions. By contrast, curly silica nanofibers have been less reported because the underlying growth mechanism remains unexplored, hindering further morphology control for applications. Herein, we describe the synthetic protocol for silica nanofibers with a tunable curliness based on the control of the water-in-oil emulsion droplets. Systematically decreasing the droplet size and increasing their contact angle, the Brownian motion of the droplets intensifies during the silica growth, thus increasing the random curliness of the nanofibers. This finding is supported by simplistic theoretical arguments and experimentally verified by varying the temperature to finely tune the curliness. Assembling these nanofibers toward porous disordered films enhances multiple scattering in the visible range, resulting in increased whiteness in contrast to films constructed by spherical and rod-like building units, which can be useful for, e.g., coatings and pigments.

## Highlights

• Silica nanofibers with well-defined randomly curly morphologies are synthesized
• Brownian-motion-induced growth mechanism of nanofibers is identified
• The mechanism guides further shape control of silica nanofibers, e.g., by temperature
• Assembling nanofibers into films allows visible light scattering for high whiteness

## Graphical abstract

**Figure:**
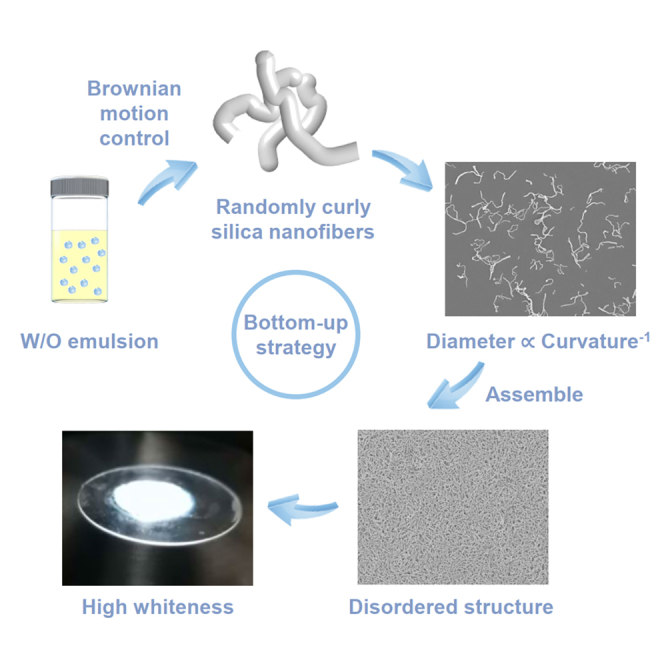

## Teaser

Lin et al. elaborate on the synthetic scope of randomly curly silica nanofibers with a precise morphology regulation. This curly morphology is found to be profoundly impacted by the Brownian motion. Assembling nanofibers toward porous disordered films enhances multiple light scattering in the visible range, resulting in distinct matte whiteness.

## Introduction

Colloidal assembly has matured into a large field, typically aiming at well-defined structural units toward self-assemblies and new functionalities.^1^^,^^2^^,^^3^ Therein, rod-like silica colloids have already provoked extensive research for, e.g., colloidal crystals,^4^ Pickering emulsifiers,^5^ field-driven assemblies,^6^ biomedical detection,^7^ bioimaging,^8^ biomarkers,^9^ and catalysis,^10^ defined by their controlled anisotropic shape. Therein, the anisotropic growth of silica nanorods has been unraveled by Kuijk et al.,^11^ where aqueous droplets of water-in-oil emulsion absorb silica precursors from their surroundings. Condensation of silica occurs anisotropically at the water-oil interface, directing the growth of a silica nanorod out from the droplet. Subsequent efforts have been devoted primarily to the precise control of the rod-like shape.^12^^,^^13^^,^^14^ By contrast, only a few investigations deal with curly worm-like silica nanofibers,^15^ wherein the underlying mechanisms are largely unexplored. Here, we show how to understand and tune the curliness of such materials to control the disorder and packing, which is useful for promoted interfacial scattering, e.g., whiteness for pigment applications.

We synthesize silica nanofibers with tuneably worm-like shapes. The curliness can be enhanced by decreasing the size of the aqueous droplets or increasing the contact angle (*θ*) of the droplets hosting the silica nanoparticles within the solvent. Essentially, the droplets undergo continuous Brownian motion during the nanofiber growth,^16^ thus facilitating the randomly curly colloidal shape. Supported by simple models, we further suggest the confirmation of this random-motion dependence by varying the reaction temperature to regulate the curliness. Finally, the worm-like nanofibers self-assemble into porous networks, lacking long-range correlations, capable of multiple scatterings of visible light and therefore leading to whiteness. In all, raising dynamic Brownian motion to randomize the growth of silica nanofiber proposes a new insight in regulating the shape of colloids. Utilizing randomly curly colloids offers new approaches for advanced broadband light scattering.

## Results and discussion

### Synthesis and morphological study of silica colloids

**Figure 1:**
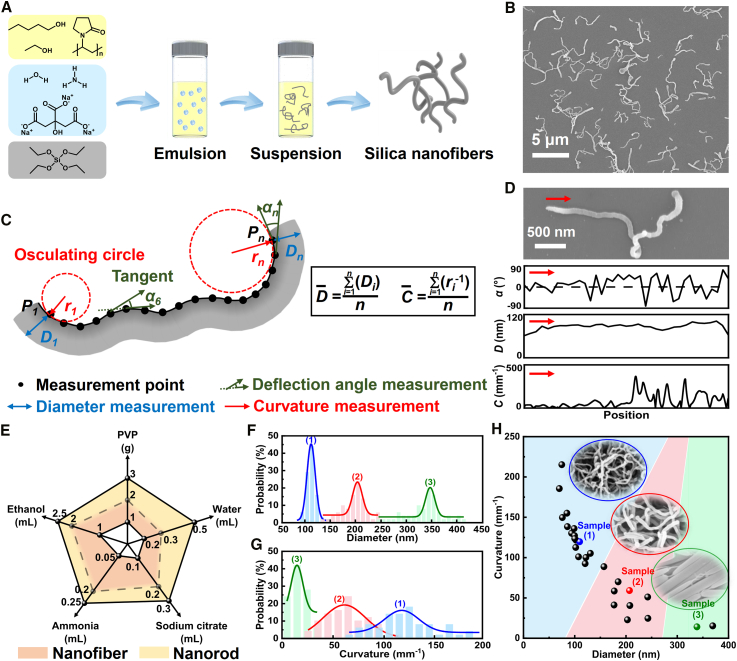
Synthesis and morphologies of anisotropic silica colloids (A) Schematics of the synthesis route. The water-in-oil emulsion is composed of pentanol, ethanol, and PVP phase (yellow) with suspensions of aqueous droplets of sodium citrate and ammonia (blue). The silica precursor (gray) condenses inside the droplets and forms nanofibers guided by the droplet thermal movement. (B) Scanning electron microscopy (SEM) observation of nanofibers. The nanofibers are synthesized with 1 g PVP, 10 mL 1-pentanol, 0.25 mL water, 0.1 mL sodium citrate aqueous solution, 0.15 mL ammonia, 2 mL ethanol, and 0.1 mL tetraethyl orthosilicate (TEOS). (C) Definitions of deflection angle *α*, diameter *D*, and curvature *C*. (D) The *α*, *D*, and *C* measured over a single nanofiber. (E) Reactant compositions leading to nanofibers and nanorods. (F and G) The shape control of nanofibers by increasing the water content from (1) 0.25 to (2) 0.3 and (3) 0.5 mL. (H) Sorting nanosilica morphologies using machine learning (K-means clustering).

In synthesis, the water-in-oil emulsions are prepared as adapted from a previous method,^11^ where aqueous droplets are suspended in pentanol and stabilized by sodium citrate and polyvinylpyrrolidone (PVP) (Figure 1A; see also the experimental procedures for details). The droplets have an average hydrodynamic diameter of ∼160 nm with relatively uniform size distribution, close to the size of the resulting nanosilica, hinting at the determining role of the droplet in defining the morphology of the products (Figure S1). First, it is qualitatively observed that depending on the reactant compositions, spheres, nanorods, curly nanofibers, and even bullet-shaped colloids are formed (see supplemental information). Within the emphasis of this work, Figures 1B and S2 show examples of the curly nanofibers, which are randomly tortuous and individually separated. The nanofiber randomness is suggestive for Brownian motion where a colloid walks stochastically in a medium^16^ and will be discussed herein. These nanofibers have relatively stable properties due to silica’s robust resistance to high temperatures and low pH.

To allow quantitative characterization, especially for the nanofibers, three structural parameters are identified, i.e., deflection angle, diameter, and curvature (Figure 1C). First, the nanofibers imaged on a substrate are assigned equidistant points (*P*_*n*_, typically *n* > 30) along them. At each *P*_*n*_, the deflection angle (*α*_*n*_) is measured as the angle change between two neighboring tangents, where the clockwise direction is ruled as positive.^17^ Similarly, the diameter (*D*_*n*_) is measured at different *P*_*n*_. The curvature (*C*_*n*_) is the amount by which a curve deviates from being a straight line.^18^ It is characterized by the reciprocal of an osculating circle’s radius (*r*_*n*_) as shown in Figure 1C (see Figure S3 and Note S1 for details). *D* and *C* are finally averaged, i.e., $\bar{D}$ and $\bar{C}$ (Figure 1C). For nanofibers, *D* remains constant, but both *α* and *C* vary randomly over nanofibers, implying that they are irregularly curly (Figures 1D and S4).

The experimentally observed shapes (Figures S5–S12) are next analyzed using the above parameters. Generally, increasing the reactant concentration and reaction time increases $\bar{D}$ but decreases $\bar{C}$ (Figures 1E and S12). For instance, increasing the water content increases $\bar{D}$ while decreasing $\bar{C}$, leading to nanorods (Figures 1F and 1G). Scaling up the synthesis conditions up to 20-fold does not affect the morphology of nanofibers (Figure S11). Structural parameters of nanofibers and nanorods are illustrated in Figure 1H. Interestingly, decreasing $\bar{D}$ (<150 nm) always leads to nanofibers instead of nanorods. Conversely, large $\bar{D}$ (>280 nm) leads to nanorods. Nanofibers with moderate diameters (150–280 nm) are coarsely curly. Next, a machine learning algorithm (K-means) is used to cluster these results (Figure S13; Table S1). Three groups are sorted as shown in Figure 1H, where blue, red, and green regions represent nanofibers with decreasing curliness.

### Divergent growth evolution of silica nanorods and nanofibers

Next, we explore the mechanisms that lead to nanofibers or nanorods. As already elaborated for the growth of silica nanorods, aqueous droplets stabilized by sodium citrate are dispersed in pentanol before adding a silica precursor.^11^ The silica precursor is hydrolyzed into water-favorable entities in an alkaline environment, which diffuses into droplets and condenses into silica. The growth of silica is anisotropic and guided by the droplet properties, leading to either straight nanorods or curly nanofibers.^11^^,^^19^

**Figure 2:**
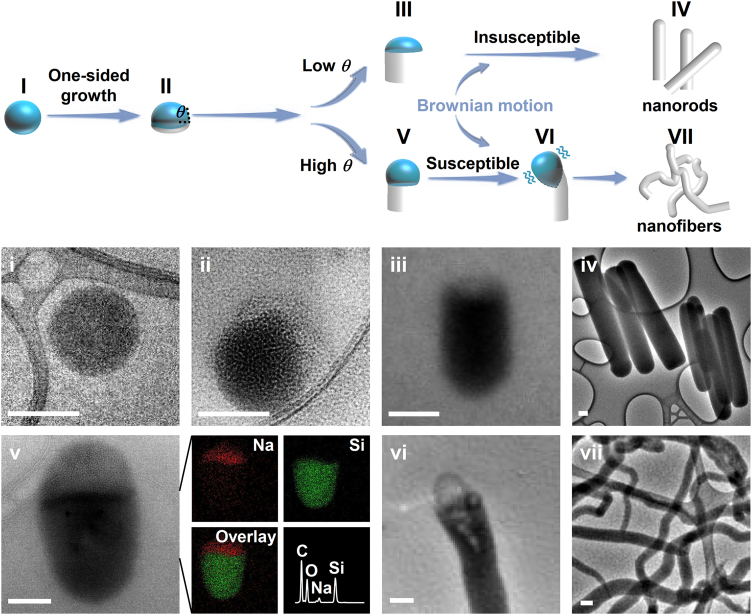
Growth evolution of silica nanorods and nanofibers Scale bars: 100 nm.

Previously, it has been suggested that curliness is caused by anisotropic interface tension.^15^ Here, we suggest another mechanism, supported by transmission electron microscopy (TEM) observation and elemental analysis (Figure 2). The emulsion droplet during the synthesis is visible by TEM due to a high concentration of sodium citrate in the droplet (elemental mapping in Figure 2). Upon evaporating the water, sodium citrate serves as a contrast agent to distinguish the silica components.

Initially, solid silica forms at one side of an emulsion droplet (Figure 2II), indicating divergent growth. The droplet anchored to a silica nanofiber shows a larger *θ* (Figure 2V) than that anchored to a nanorod (Figure 2III). This discrepancy is also verified at macroscopic length scale (Figures S14 and S15), where a water droplet stabilized by sodium citrate sits on a silica wafer within PVP-dissolved pentanol. Microscopically, colloidal droplets with a large *θ* are susceptible to Brownian motion^20^^,^^21^ compared to those with a small *θ*.^22^ In contrast, the influence of Brownian motion on nanosilica is far weaker than that on the droplets because of the significantly larger longitudinal size and larger density of the nanosilica compared to the droplets (see Note S2 for details). Thus, for the sake of simplicity, we exclude the impact of nanosilica motion in the following analysis. The stochastic thermal perturbations to droplets are sustained over the whole growth, allowing randomly curly morphology (Figures 2V–2VII). Furthermore, a large *θ* dictates large interfaces between a droplet and its ambiance to enrich the silica precursor condensing at the edges, causing heterogeneous growth across nanofibers, i.e., appearing as V-shaped cavities (Figure 2VI).

In all, the silica precursor hydrolyzes and diffuses into a droplet. It accumulates within one side of the droplet, becoming silica particles. The droplet, while attached to the silica, plays a role in guiding the growth of these particles. It undergoes continuous stochastic thermal perturbation, leading to the formation of randomly curly nanofibers until the depletion of the silica precursor.

### The suspension droplet effect on the silica morphology

**Figure 3:**
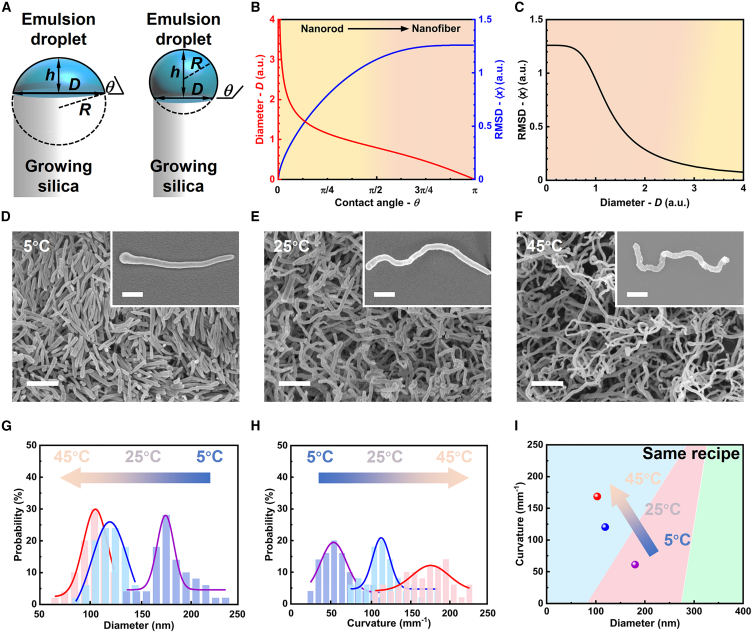
Modeling and experimental results toward rationalizing the suspension droplet effect on the silica growth (A) A schematic spherical cap mode for simulating a droplet template. (B) The theoretical relationships between basal area *D*/Brownian motion root-mean-square displacement (RMSD) (⟨*x*⟩) and the contact angle (θ) of droplet on silica in solvent. (C) The derived relationships of *D* and ⟨*x*⟩. (D–F) Temperature-regulated morphology of nanofibers synthesized using the same recipe as Figure 1B at (D) 5°C, (E) 25°C, and (F) 45°C. Scale bars: 2 μm and 500 nm in the insets. (G and H) The *D* and the *C* control by temperatures. (I) Experimental verification of the morphology diagram produced using machine learning.

To further explore the potential correlation of Brownian motion and nanofiber shape, we introduce a simple model using a spherical cap for the emulsion droplet templated at the edge of the growing colloid (Figure 3A). Given a constant droplet volume, the sphere’s radius (*R*) and the basal nanofiber diameter (*D*) rely on the *θ* of the droplet on silica within solvent^23^^,^^24^:

(Equation 1) $$R=A*{\left(2-3\;\cos\;\theta +\cos^{3}\;\theta \right)}^{-\frac{1}{3}}$$

(Equation 2) $$D=2*A*{\left(2-3\;\cos\;\theta +\cos^{3}\;\theta \right)}^{-\frac{1}{3}}*\sin\;\theta$$

where *A* is a geometrical constant (see Note S2 for detailed derivation). Brownian motion is a form of random walk process; thus, it can be simplistically described by the Stokes-Einstein-Sutherland equation, where the root-mean-square displacement (⟨*x*⟩) of the droplet undergoing a random walk in a solvent is both *R* and *θ* dependent^25^^,^^26^:

(Equation 3) $$\left\langle x\right\rangle ={\left(\frac{k_{B}Tt}{3\pi \eta R}\right)}^{\frac{1}{2}}\propto {\left(2-3\;\cos\;\theta +\cos^{3}\;\theta \right)}^{\frac{1}{6}}$$

where *k*_*B*_ is the Boltzmann’s constant, *T* is the absolute temperature, *η* is the dynamic viscosity, and *t* is the time (see Note S2 for detailed derivation).

As shown in Figure 3B, increasing *θ* decreases *D*, while it increases the ⟨*x*⟩ (Equations 2 and 3), signifying that smaller droplets are more susceptible to stochastic thermal movement. These results are in concert with the experimental results of Figures 2 and S16, corroborating again that increasing *θ* and decreasing *D* facilitate curly morphology. Explicitly, the ⟨*x*⟩ shows the negative correlation to the *D* (Figure 3C; see Note S2 for detailed derivation), in concert with the experimental observation (also negative correlation in Figure 1H), where the *C* is considered instead of the ⟨*x*⟩. This unveils the positive correlation of curly morphology and Brownian motion.

To find still more direct proof that thermally driven Brownian motion would control the curling, curliness versus the synthesis temperature is followed. Upon heating, the droplet size diminishes due to the increased solubility of aqueous droplets in pentanol,^19^ and Brownian motions are promoted (Equation 3).^27^ Indeed, elevating the synthesis temperature increases the curvature while thinning the nanofibers, as qualitatively illustrated in Figures 3D–3F and more quantified in Figures 3G and 3H. This again corroborates the theory and suggests temperature as a valuable way for morphological control besides varying the reactant contents (Figure 3I).

### Application for whiteness

**Figure 4:**
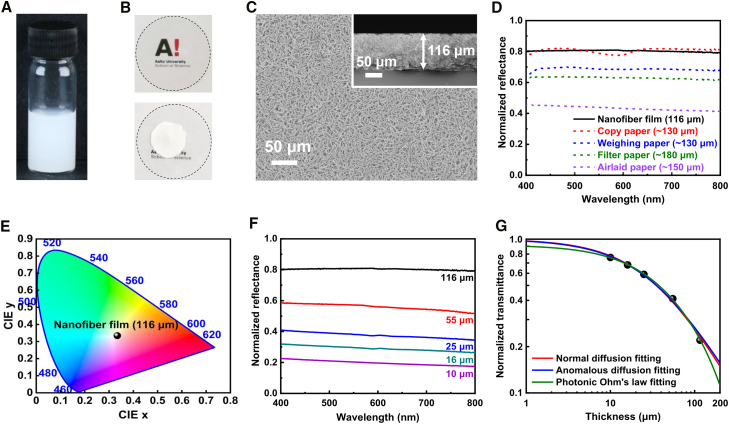
Whiteness characterization of the film constructed by nanofibers with an average *D* of 110 nm and an average *C* of 120 mm^−1^ (A) Photograph of nanofiber dispersion in ethanol (10 mg mL^−1^). (B) Photographs of a dried nanofiber film on a glass substrate (bottom) in comparison to a bare glass substrate (top). (C) Surface and cross-sectional (inset) SEM observation of the film. (D) Normalized total reflectance spectra of a 116-μm-thick nanofiber film in contrast to commercial papers. (E) CIE chromaticity coordinate of 116-μm-thick nanofiber film. (F) Normalized total reflectance spectra of nanofiber films with different thicknesses. (G) Fittings of normalized transmittance with three models, i.e., normal diffusion, anomalous diffusion, and photonic Ohm’s law.

Inspired by the whiteness of *Cyphochilus* beetle^28^^,^^29^ and classical paper sheets^30^ based on their disordered micro-architectures that enable remarkable optical scattering, herein we constitute randomly structured films, allowing high whiteness based on the curly silica nanofibers. This is characterized by sedimenting nanofibers from ethanol on a glass slide and then drying them naturally (Figures 4A, 4B, and S17).

Interestingly, the coating is homogeneous in the structure, independent of its thickness (Figures 4C and S17), probably because the nondirective capillary forces induced among randomly curly nanofibers ameliorate the coffee ring effect.^31^ Notably, the structural whiteness is a result of omnidirectional optical scattering caused by the randomly woven nanofiber structure. It is highly porous, grossly increasing the silica-air interfaces, thus facilitating multiple visible light scatterings to allow whiteness (Figures 4C and S18).^32^

Next, we investigate the optical property of nanofiber films in comparison to commercial papers. The total reflectance of a 116-μm-thick film shows a broadband (400–800 nm) reflectance higher than 0.8, outperforming those of commercial papers with a comparable/larger thickness (Figures 4D and S19). This potent visible light scattering enables matte whiteness (Figure 4B), as also evidenced by the International Commission on Illumination (CIE) chromaticity coordinate analysis (Figure 4E).

Eventually, to access the intrinsic light scattering properties of films, we applied three models, i.e., normal diffusion, anomalous diffusion, and photonic Ohm’s law,^29^^,^^30^^,^^33^ to fit their transmittance at different thicknesses (see Note S3 for details). Reducing the film thickness deteriorates the broadband reflectance while increasing the transmittance (Figures 4F and 4G). All of the three models fit well (Table S2). Among them, the anomalous diffusion model approximates the normal diffusion model because the exponents in two models are almost identical (Table S2). Moreover, in the photonic Ohm’s law model, the parameter describing the absorption effect approaches zero, indicating the low visible light absorption of films (Table S2; Note S3). Overall, the light scattering of films can be well described by the normal diffusion model with almost no absorption.

### Whiteness comparison

**Figure 5:**
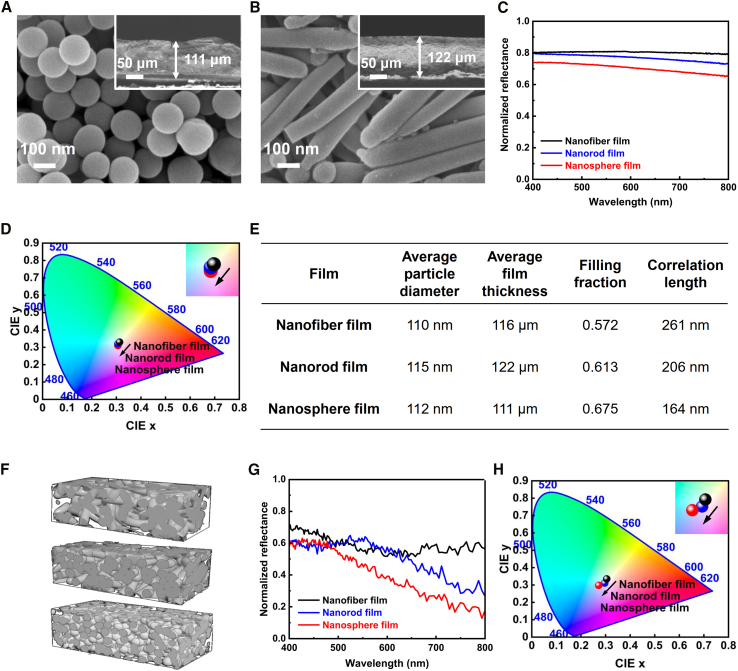
Whiteness performance of the films from the differently shaped building units (A and B) Surface and cross-sectional (inset) SEM observation of (A) the nanosphere film and (B) the nanorod film. (C) Normalized total reflectance spectra of nanofiber, nanorod, and nanosphere films. (D) CIE chromaticity coordinate of the films. (E) Summarized parameters of the films obtained from the SEM cross-sectional view of the films in Figure S20. (F) Representative sketches of models. From top to bottom: nanofiber film, nanorod film, and nanosphere film. (G) Simulated total reflectance spectra of the films. (H) CIE chromaticity coordinate of the films with the simulated spectra.

Next, we compare the light-scattering performance of films constructed by differently shaped building blocks. By contrast, silica nanospheres (Figure 5A) and nanorods (Figure 5B) with comparable diameters to nanofibers are used, assembling into films at thicknesses equivalent to those of nanofiber film (Figures 4C, 5A, 5B, and 5E). Among the films, the nanofiber one shows the highest total reflectance over visible light regions (Figure 5C) despite having the lowest filling fraction (Figure 5E). The reflectance of the nanofiber film is uniform across 400–800 nm, indicating an ideal white color appearance. In contrast, the reflectance of both nanosphere and nanorod films decreases in the long wavelength regions, suggesting a nonideal visible light reflection, i.e., a slightly blueish appearance (Figure 5D). This particular defect may impact applications with a demand for high-quality whiteness, but it can be minimized if using the curly nanofiber film.

To validate the experimental findings, we have conducted finite-difference time domain (FDTD) simulations. Based on the parameters shown in Figure 5E, three models have been established as sketched in Figure 5F. Herein, the filling fraction is the effect volume fraction of nanoparticles within the films. The correlation length is a statistical and physical characteristic scale that describes the randomness or disorder degree in a system. It refers to the distance within which physical quantities of particles exhibit correlation.^34^ Both of them are calculated from the cross-sectional view of the films (Figure S20). The simulation reflectance follows the order of nanofiber film > nanorod film > nanosphere film across most wavelengths (Figure 5G), consistent with the experimental results. Similarly, the reflectance of the nanofiber film remains flat, while the others decay with an increasing wavelength. This corroborates the chromatic observations (Figure 5H). The discrepancy in intensity between the simulation and experimental spectra can be attributed to the filling fraction of nanosilica, where the filling fraction used for simulation is obtained from 2D cross-sectional images, which may deviate from the value of global 3D structures.

In practice, liquids, e.g., water and oils, favor wetting the nanofiber film (Figure S21), deteriorating the whiteness due to the refractive index matching. Modification with a hydrophobic silane allows film for liquid repellence, preserving whiteness (Figure S21). Thus, it promises great commercial potentials as, e.g., coatings and pigments.

In summary, we describe a synthetic protocol and mechanistic understanding for silica nanofibers with randomly curly morphology via water-in-oil emulsions where aqueous droplets are the template, allowing the construction of disordered architectures with whiteness. During the synthesis, the Brownian motion of droplets allows the stochastic growth of nanofibers, leading to worm-like shapes. Tuning the intrinsic size and wetting nature of aqueous droplets sitting on silica within solvents is crucial for curly morphology dominated by thermal dynamic fluctuation. Assembling nanofibers into disordered films allows for broadband visible light scattering in a normal diffusion mode, resulting in matte whiteness. This work suggests a unique dynamic design of disorderly shaped colloids for constructing white materials. Additionally, incorporating a secondary phase, e.g., magnetic or dielectric materials, into silica nanofibers allows for on-demand properties and the assembly of composite nanofibers, enabling add-on functions.^35^^,^^36^^,^^37^

## Experimental procedures

### Resource availability

#### Lead contact

Further information and requests for resources and reagents should be directed to and will be fulfilled by the lead contact, Bo Peng (pengbo006@gmail.com).

#### Materials availability

All materials used in this work are commercially available.

#### Data and code availability

All data supporting the findings of this study are available in the article and supplemental information or from the lead contact upon request.

### Synthesis and characterization of nanofibers

1 g PVP (average molecular weight [*M*_*n*_] = 40,000 g mol^−1^) was dissolved in 10 mL 1-pentanol by sonication. Afterward, 0.25 mL water, 0.1 mL sodium citrate aqueous solution (0.18 mol L^−1^), 0.15 mL ammonia, and 2 mL ethanol were added to the pentanol solution, followed by violent shaking and sonication for 1 min to form a well-dispersed emulsion. Then, 0.1 mL tetraethyl orthosilicate was added dropwise into the emulsion and mixed by slight shaking and sonication for 10 s. All mixtures were left to rest for reaction at room temperature for 6 h. After the reaction, the turbid mixtures were centrifuged at 1,500*g* for 30 min. The supernatant was removed, while the sediment was redispersed in the ethanol by sonication. This particle dispersion was centrifuged at 700*g* for 15 min, followed by a new dispersive process with water and then centrifugation. The whole centrifugation procedure was repeated at least 3 times to remove the reactant residue. After separation, the samples were dried naturally, and the white powder of the silica nanofibers was finally yielded. The aforementioned reaction condition was defined as the standard condition. Lastly, the morphology of silica nanofibers was studied by scanning electron microscopy (SEM; Zeiss Sigma VP), TEM (JEOL JEM-2200FS), and atomic force microscopy (Bruker Dimension Icon) with a tapping mode. ImageJ (v.1.53) was used to characterize the morphological parameters of nanofibers.

### Systematic experiments for morphological control

To study the influence of reaction conditions on the morphology of final products, six variable experimental parameters were varied, including the amounts of PVP (0.5, 1, 1.5, 2, 3, and 5 g), water (0.05, 0.1, 0.15, 0.2, 0.25, 0.3, 0.5, 0.75, and 1 mL), sodium citrate (0.05, 0.1, 0.15, 0.2, 0.25, and 0.3 mL), ammonia (0.05, 0.1, 0.15, 0.2, 0.25, and 0.5 mL), ethanol (0.5, 1, 1.5, 2, 2.5, and 3 mL), and reaction time (3, 6, 9, 12, 24, and 48 h). When a variable was studied, the other variables remained as the standard condition. Moreover, as for large-scale reactions, the reagents were scaled up at the given ratio (5-, 10-, and 20-fold) of the standard condition.

### Preparation, characterization, and scattering performances of nanofiber films

Sedimentation was used for preparing nanofiber films. 10 mg silica nanofiber powder was dispersed in 1 mL ethanol by sonication. A filter instrument (Millipore) was assembled with thin rounded glass sheets (Menzel Glaser, diameter: 25 mm) as the substrates. The casting solution was carefully added into the filter cup, and then the whole filter was kept vertical until the ethanol evaporated totally. White films were finally acquired as nanofibers deposited onto the surfaces of glass substrates. Films with different thicknesses were prepared in the same way but with different nanofiber concentrations. Moreover, the films made by nanospheres and nanorods were produced adopting the same process.

Next, the surface and cross-sectional structures of films were observed by SEM. For cross-sectional observation, the samples were prepared by cryo-fracturing the films in liquid nitrogen. About 4-nm-thick platinum layers were coated on the surface of samples before observation using a sputtering coater (Leica EM ACE600). In addition, the other SEM (JEOL JIB-4700F) with a focused ion beam module was used to mill the flat and smooth cross-sectional areas within films to acquire basic parameters for modeling.

Lastly, the ultraviolet-visible total reflectance and transmittance spectra (400–800 nm) of the films were measured with an ultraviolet-visible spectrophotometer (Agilent Cary 5000) with an integrating sphere (Agilent DRA 2500). All films were tested under the same environmental conditions.

### FDTD simulations

After getting cross-sectional SEM images, ImageJ software was used for thresholding of the images and then calculating the filling fraction (*V*_*f*_). The characteristic length scale of the structures (*I(x)*) was determined by radially averaged two-point correlation function using the fast Fourier transform (FFT) method as follows:

(Equation 4) $$S_{2}\left(r\right)=\frac{\sum_{l,m,n\in \Omega }{\text{FFT}}^{-1}\left({\left|\text{FFT}\left(I\left(x\right)\right)\right|}^{2}\right)}{\omega }$$

where $\Omega =\left\{\left(l,m,n\right)|l^{2}+m^{2}+n^{2}=r^{2},r\leq N/2\right\}$ and *ω* is the number of elements in *Ω*. Then, the correlation length (*l*_*c*_) was defined as the position where the radially averaged two-point correlation function has its minimum:

(Equation 5) $$l_{c}={\mathrm{argmin}}_{r}{\;S}_{2}\left(r\right)$$

Afterward, the models could be built, and FDTD simulations were carried out by using Lumerical software (Ansys Canada 2020a-r5). Boundary conditions were set as periodic with perfect matching layer boundaries in both the x and y directions. The wavelength range of the broadband source was set from 400 to 800 nm in p-polarization coming from the vertical direction. The refractive index of silica materials was set as 1.45 in all cases. The numerical stability and convergence were ensured with the adequate boundary condition, and the simulations were carried out until all incoming light had either reflected or transmitted.

## Supplemental information

Document S1. Figures S1‒S21, Tables S1 and S2, and Notes S1‒S3

Document S2. Article plus supplemental information

## References

[1] Whitesides G.M., Grzybowski B. (2002). Self-assembly at all scales. Science.

[2] Grzelczak M., Vermant J., Furst E.M., Liz-Marzán L.M. (2010). Directed self-assembly of nanoparticles. ACS Nano.

[3] El-Shater R.E., El Shimy H., Saafan S.A., Darwish M.A., Zhou D., Trukhanov A.V., Trukhanov S.V., Fakhry F. (2022). Synthesis, characterization, and magnetic properties of Mn nanoferrites. J. Alloys Compd..

[4] Liu B., Besseling T.H., Hermes M., Demirörs A.F., Imhof A., Van Blaaderen A. (2014). Switching plastic crystals of colloidal rods with electric fields. Nat. Commun..

[5] Kim K., Kim S., Ryu J., Jeon J., Jang S.G., Kim H., Gweon D.-G., Im W.B., Han Y., Kim H., Choi S.Q. (2017). Processable high internal phase Pickering emulsions using depletion attraction. Nat. Commun..

[6] Yan J., Chaudhary K., Chul Bae S., Lewis J.A., Granick S. (2013). Colloidal ribbons and rings from Janus magnetic rods. Nat. Commun..

[7] Zhang P., Zhou X., He M., Shang Y., Tetlow A.L., Godwin A.K., Zeng Y. (2019). Ultrasensitive detection of circulating exosomes with a 3D-nanopatterned microfluidic chip. Nat. Biomed. Eng..

[8] Shimoni O., Yan Y., Wang Y., Caruso F. (2013). Shape-dependent cellular processing of polyelectrolyte capsules. ACS Nano.

[9] Xu C., Niu Y., Popat A., Jambhrunkar S., Karmakar S., Yu C. (2014). Rod-like mesoporous silica nanoparticles with rough surfaces for enhanced cellular delivery. J. Mater. Chem. B.

[10] Yi D., Zhang Q., Liu Y., Song J., Tang Y., Caruso F., Wang Y. (2016). Synthesis of chemically asymmetric silica nanobottles and their application for cargo loading and as nanoreactors and nanomotors. Angew. Chem. Int. Ed..

[11] Kuijk A., Van Blaaderen A., Imhof A. (2011). Synthesis of monodisperse, rodlike silica colloids with tunable aspect ratio. J. Am. Chem. Soc..

[12] He J., Yu B., Hourwitz M.J., Liu Y., Perez M.T., Yang J., Nie Z. (2012). Wet-chemical synthesis of amphiphilic rodlike silica particles and their molecular mimetic assembly in selective solvents. Angew. Chem. Int. Ed..

[13] Hagemans F., Pujala R.K., Hotie D.S., Thies-Weesie D.M.E., de Winter D.A.M., Meeldijk J.D., van Blaaderen A., Imhof A. (2019). Shaping silica rods by tuning hydrolysis and condensation of silica precursors. Chem. Mater..

[14] Murphy R.P., Hong K., Wagner N.J. (2017). Synthetic control of the size, shape, and polydispersity of anisotropic silica colloids. J. Colloid Interface Sci..

[15] Yi D., Xu C., Tang R., Zhang X., Caruso F., Wang Y. (2016). Synthesis of discrete alkyl-silica hybrid nanowires and their assembly into nanostructured superhydrophobic membranes. Angew. Chem. Int. Ed..

[16] Mörters P., Peres Y. (2010).

[17] Anderson I.M., Bezdek J.C. (1984). Curvature and tangential deflection of discrete arcs: A theory based on the commutator of scatter matrix pairs and its application to vertex detection in planar shape data. IEEE Trans. Pattern Anal. Mach. Intell..

[18] Mary H., Brouhard G.J. (2019). Kappa (κ): analysis of curvature in biological image data using B-splines. bioRxiv.

[19] Datskos P., Sharma J. (2014). Synthesis of segmented silica rods by regulation of the growth temperature. Angew. Chem. Int. Ed..

[20] Brown R. (1828). XXVII. A brief account of microscopical observations made in the months of June, July and August 1827, on the particles contained in the pollen of plants; and on the general existence of active molecules in organic and inorganic bodies. Phil. Mag..

[21] Hassan P.A., Rana S., Verma G. (2015). Making sense of Brownian motion: colloid characterization by dynamic light scattering. Langmuir.

[22] Danov K., Aust R., Durst F., Lange U. (1995). Influence of the surface viscosity on the hydrodynamic resistance and surface diffusivity of a large Brownian particle. J. Colloid Interface Sci..

[23] Vafaei S., Podowski M.Z. (2005). Analysis of the relationship between liquid droplet size and contact angle. Adv. Colloid Interface Sci..

[24] Letellier P., Mayaffre A., Turmine M. (2007). Drop size effect on contact angle explained by nonextensive thermodynamics. Young's equation revisited. J. Colloid Interface Sci..

[25] Uhlenbeck G.E., Ornstein L.S. (1930). On the theory of the Brownian motion. Phys. Rev..

[26] Einstein A. (1956).

[27] Rings D., Schachoff R., Selmke M., Cichos F., Kroy K. (2010). Hot brownian motion. Phys. Rev. Lett..

[28] Vukusic P., Hallam B., Noyes J. (2007). Brilliant whiteness in ultrathin beetle scales. Science.

[29] Syurik J., Jacucci G., Onelli O.D., Hölscher H., Vignolini S. (2018). Bio-inspired highly scattering networks via polymer phase separation. Adv. Funct. Mater..

[30] Toivonen M.S., Onelli O.D., Jacucci G., Lovikka V., Rojas O.J., Ikkala O., Vignolini S. (2018). Anomalous-diffusion-assisted brightness in white cellulose nanofibril membranes. Adv. Mater..

[31] Mampallil D., Eral H.B. (2018). A review on suppression and utilization of the coffee-ring effect. Adv. Colloid Interface Sci..

[32] Jacucci G., Schertel L., Zhang Y., Yang H., Vignolini S. (2021). Light management with natural materials: from whiteness to transparency. Adv. Mater..

[33] Caixeiro S., Peruzzo M., Onelli O.D., Vignolini S., Sapienza R. (2017). Disordered cellulose-based nanostructures for enhanced light scattering. ACS Appl. Mater. Interfaces.

[34] Jiao Y., Stillinger F.H., Torquato S. (2007). Modeling heterogeneous materials via two-point correlation functions: Basic principles. Phys. Rev. E.

[35] Hussein M.M., Saafan S.A., Abosheiasha H.F., Kamal A.A., Mahmoud A.E.R., Zhou D., Trukhanov S.V., Zubar T.I., Trukhanov A.V., Darwish M.A. (2023). Structural and dielectric characterization of synthesized nano-BSTO/PVDF composites for smart sensor applications. Mater. Adv..

[36] Peng B., Zhang X., Aarts D.G.A.L., Dullens R.P.A. (2018). Superparamagnetic nickel colloidal nanocrystal clusters with antibacterial activity and bacteria binding ability. Nat. Nanotechnol..

[37] Liu X., Tan H., Rigoni C., Hartikainen T., Asghar N., van Dijken S., Timonen J.V.I., Peng B., Ikkala O. (2022). Magnetic field-driven particle assembly and jamming for bistable memory and response plasticity. Sci. Adv..

