# Supplementary material for: Randomizing the growth of silica nanofibers for whiteness
Source: Cell Rep Phys Sci. 2024 Jun 19;5(6):102021. doi: 10.1016/j.xcrp.2024.102021 (PMC11211975; doi:10.1016/j.xcrp.2024.102021)
Supplement: Document S1. Figures S1‒S21, Tables S1 and S2, and Notes S1‒S3 [file mmc1.pdf]

Cell Reports Physical Science, Volume *5*

Supplemental information

Randomizing the growth of silica nanofibers for whiteness

Zhen Lin, Johannes S. Haataja, Xichen Hu, Xiaodan Hong, Olli Ikkala, and Bo Peng

Supplemental Information

The PDF file including:

Supplemental figures S1 to S21

Supplemental tables S1 to S2

Supplemental notes S1 to S3

Supplemental references S1 to S12

Supplemental Figures

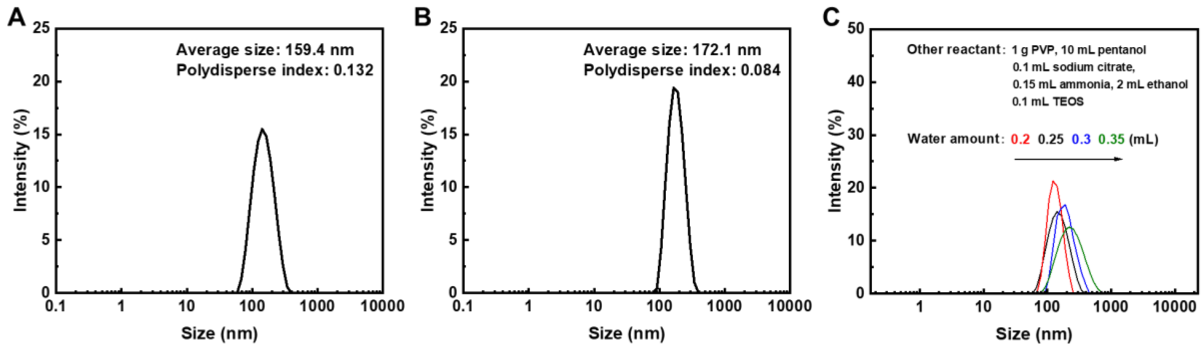

Figure S1. Size distribution of droplets in the dispersion characterized by dynamic light scattering. (A) Using the synthesis recipe of nanofibers. (B) Using the synthesis recipe of nanorods. (C) The influence of water amount on droplet size, which are consistent with the results shown in Figures 1E and S6.

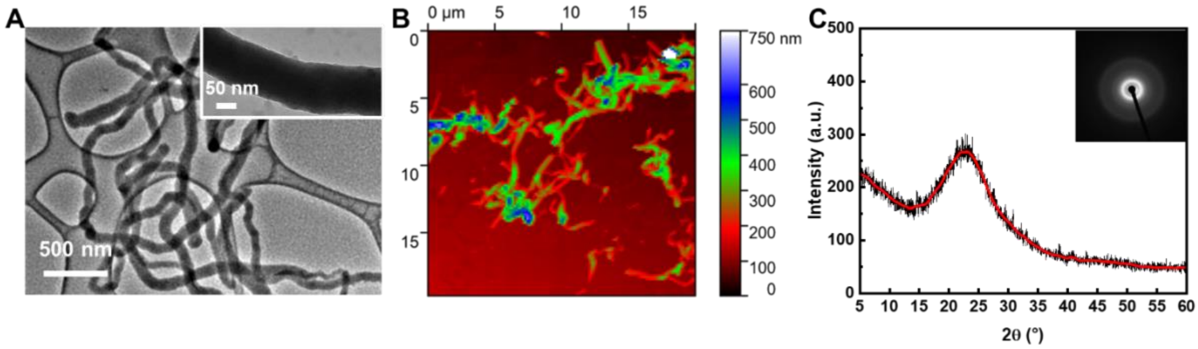

Figure S2. Characterization of nanofibers. (A) Transmission electron microscopy (TEM) images. (B) Atomic force microscopy (AFM) image. (C) X-ray diffractometer (XRD) pattern with selected area electron diffraction image (inset).

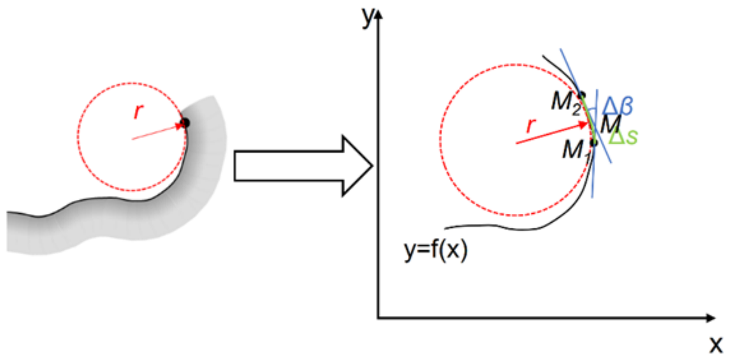

Figure S3. Schematic illustration of the curvature measurements.

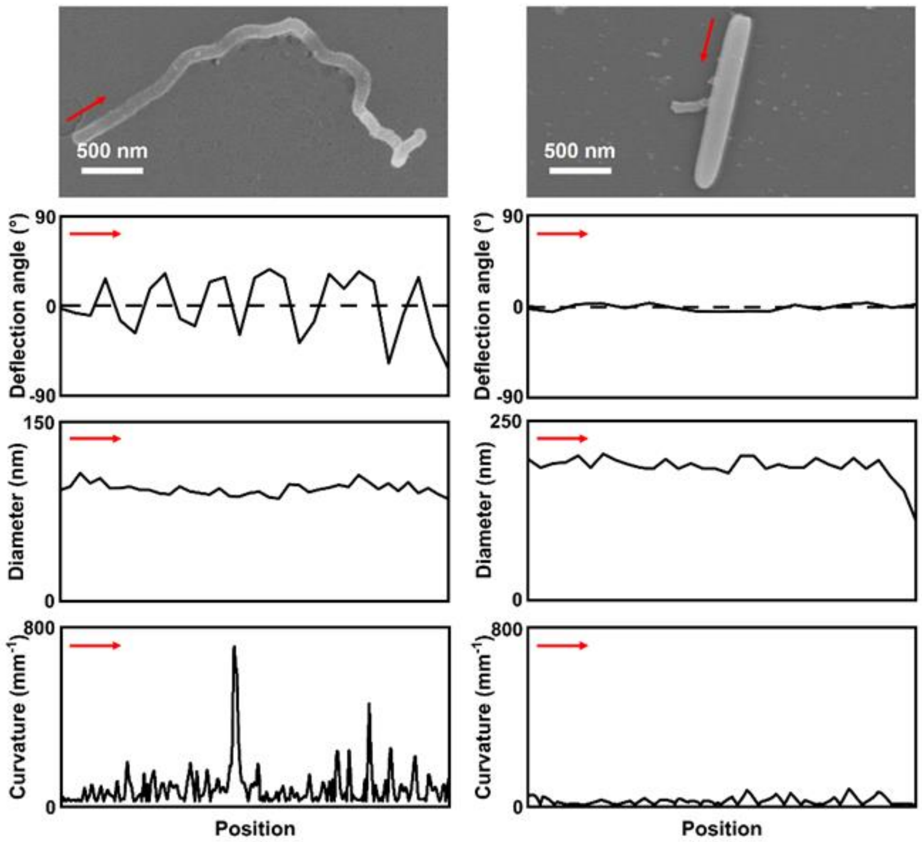

Figure S4. A single nanofiber and nanorod with in-situ deflection angle, diameter, and curvature measurement along the red arrows.

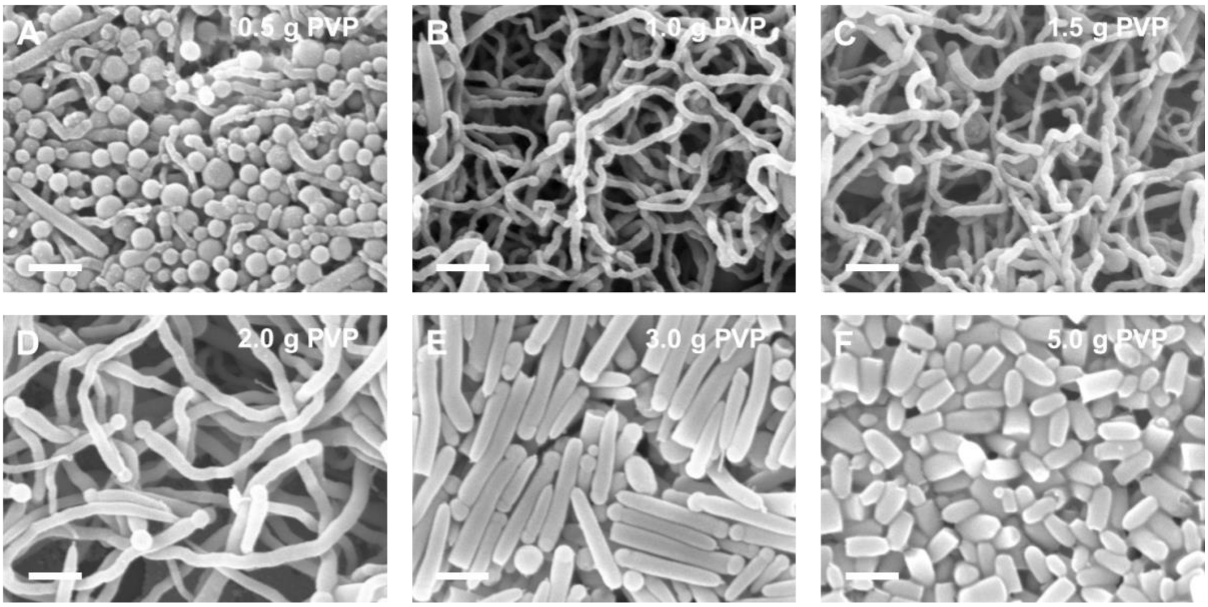

Figure S5. SEM images of silica nanoparticles prepared by different amount of PVP content. (A) 0.5, (B) 1.0, (C) 1.5, (D) 2.0, (E) 3.0, and (F) 5.0 g. The other experimental conditions are fixed as: 10 mL 1-pentanol, 0.25 mL water, 0.1 mL sodium citrate aqueous solution, 0.15 mL ammonia, 2 mL ethanol, 0.1 mL tetraethyl orthosilicate (TEOS), and 6 h growth time. The scale bars are 500 nm.

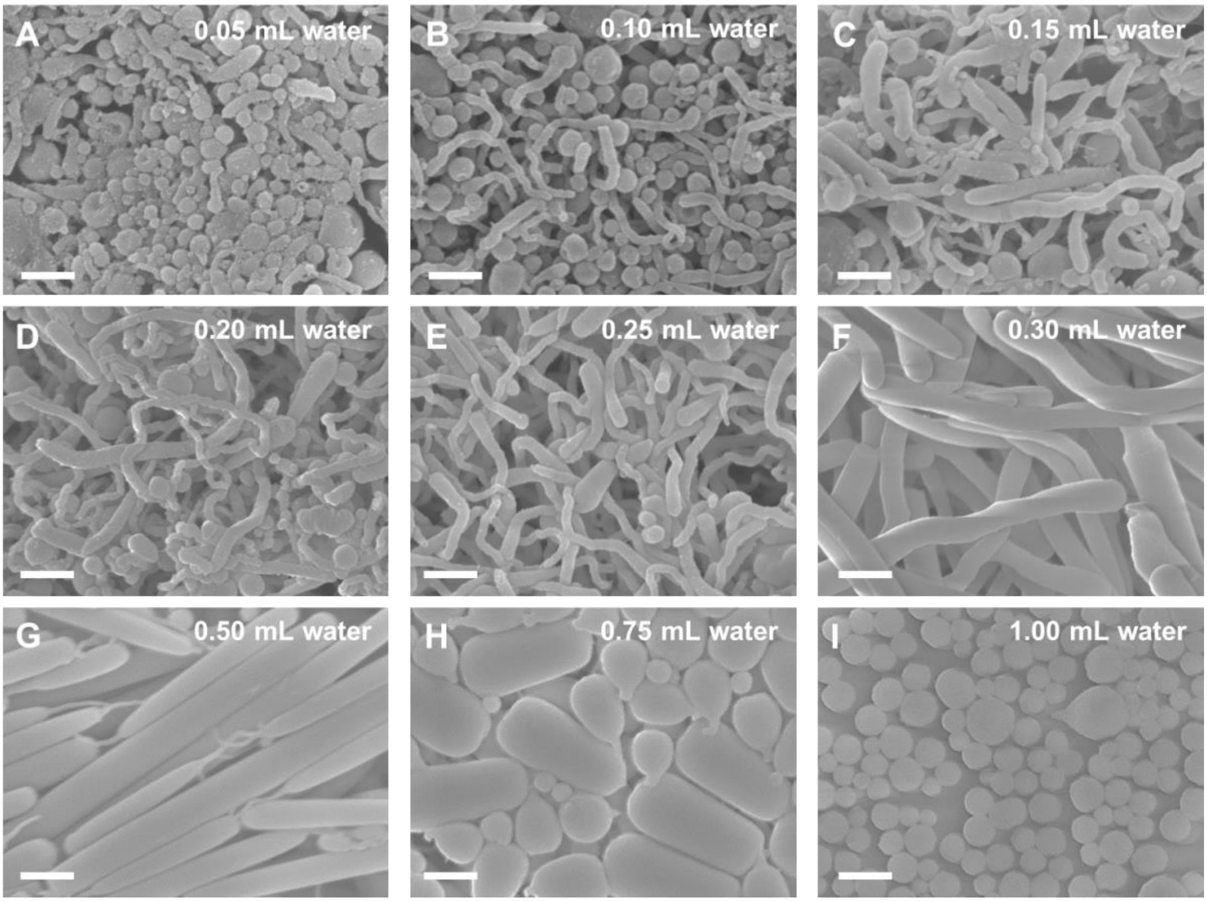

Figure S6. SEM images of silica nanoparticles prepared by different amount of water content. (A) 0.05, (B) 0.10, (C) 0.15, (D) 0.20, (E) 0.25, (F) 0.30, (G) 0.50, (H) 0.75, and (I) 1.00 mL. The other experimental conditions are fixed as: 1 g PVP, 10 mL 1-pentanol, 0.1 mL sodium citrate aqueous solution, 0.15 mL ammonia, 2 mL ethanol, 0.1 mL TEOS, and 6 h growth time. The scale bars are 500 nm.

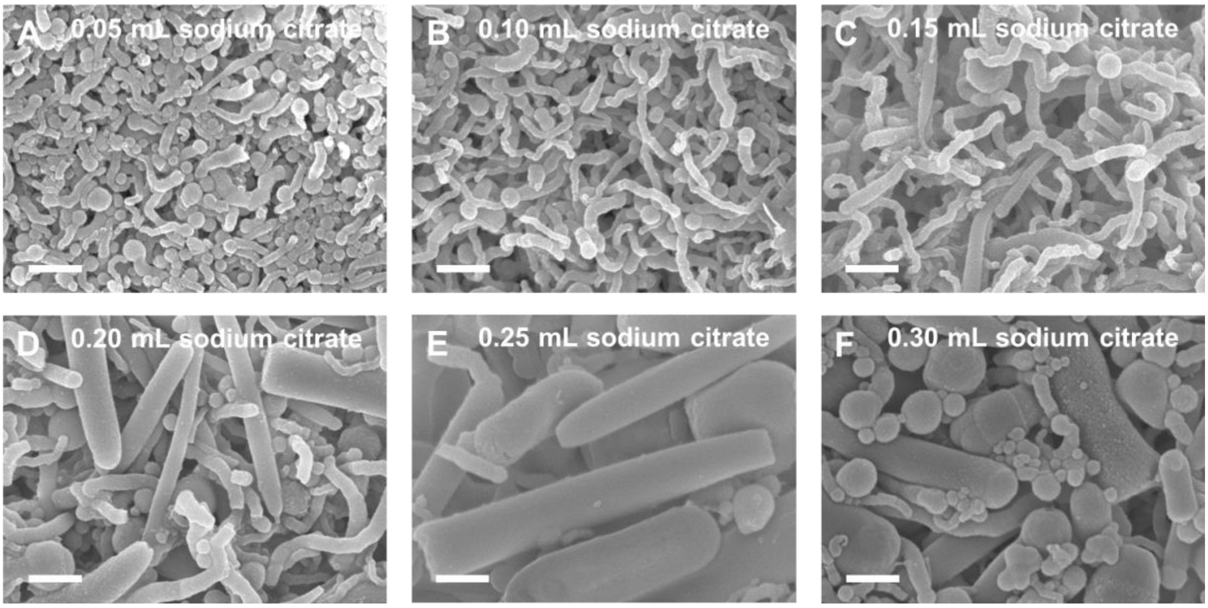

Figure S7. SEM images of silica nanoparticles prepared by different amount of sodium citrate content. (A) 0.05, (B) 0.10, (C) 0.15, (D) 0.20, (E) 0.25, and (F) 0.30 mL. The other experimental conditions are fixed as: 1 g PVP, 10 mL 1-pentanol, 0.25 mL water, 0.15 mL ammonia, 2 mL ethanol, 0.1 mL TEOS, and 6 h growth time. The scale bars are 500 nm.

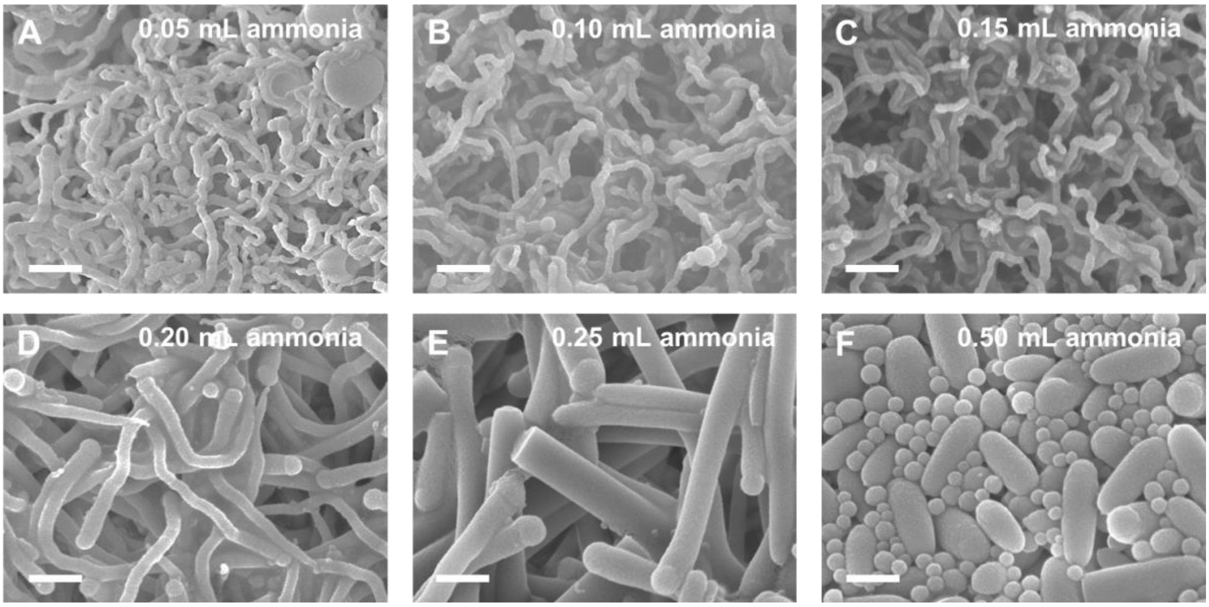

Figure S8. SEM images of silica nanoparticles prepared by different amount of ammonia content. (A) 0.05, (B) 0.10, (C) 0.15, (D) 0.20, (E) 0.25, and (F) 0.50 mL. The other experimental conditions are fixed as: 1 g PVP, 10 mL 1-pentanol, 0.25 mL water, 0.1 mL sodium citrate aqueous solution, 2 mL ethanol, 0.1 mL TEOS, and 6 h growth time. The scale bars are 500 nm.

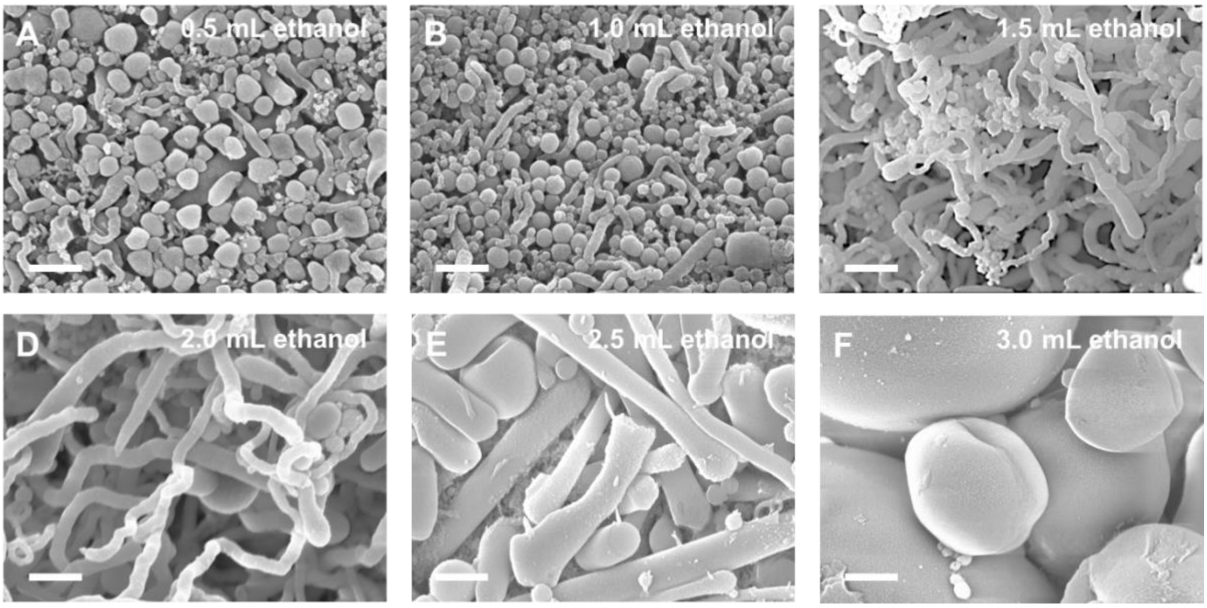

Figure S9. SEM images of silica nanoparticles prepared by different amount of ethanol content. (A) 0.5 mL, (B) 1.0 mL, (C) 1.5 mL, (D) 2.0 mL, (E) 2.5 mL and (F) 3.0 mL. The other experimental conditions are fixed as: 1 g PVP, 10 mL 1-pentanol, 0.25 mL water, 0.1 mL sodium citrate aqueous solution, 0.15 mL ammonia, 0.1 mL TEOS, and 6 h growth time. The scale bars are 500 nm.

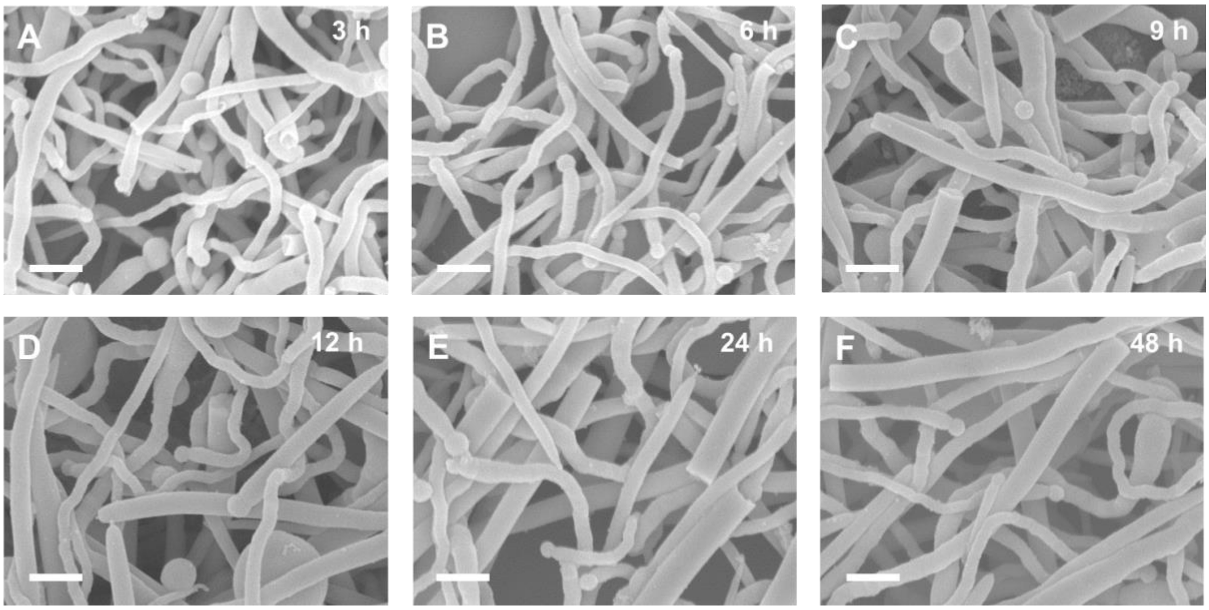

Figure S10. SEM images of silica nanoparticles observed at different growth time. (A) 3, (B) 6, (C) 9, (D) 12, (E) 24, and (F) 48 h. The other experimental conditions are fixed as: 1 g PVP, 10 mL 1-pentanol, 0.25 mL water, 0.1 mL sodium citrate aqueous solution, 0.15 mL ammonia, 2 mL ethanol, and 0.1 mL TEOS. The scale bars are 500 nm.

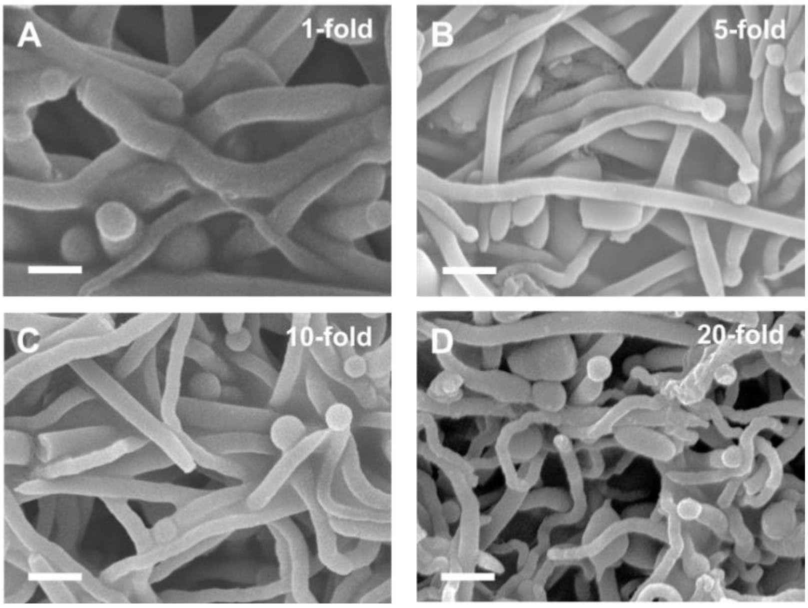

Figure S11. SEM images of silica nanoparticles prepared in the large-scale synthesis. (A) 1-fold, (B) 5-fold, (C) 10-fold and (D) 20-fold. The recipe for 1-fold synthesis is: 1 g PVP, 10 mL 1-pentanol, 0.25 mL water, 0.1 mL sodium citrate aqueous solution, 0.15 mL ammonia, 2 mL ethanol, and 0.1 mL TEOS. The scale bars are 500 nm.

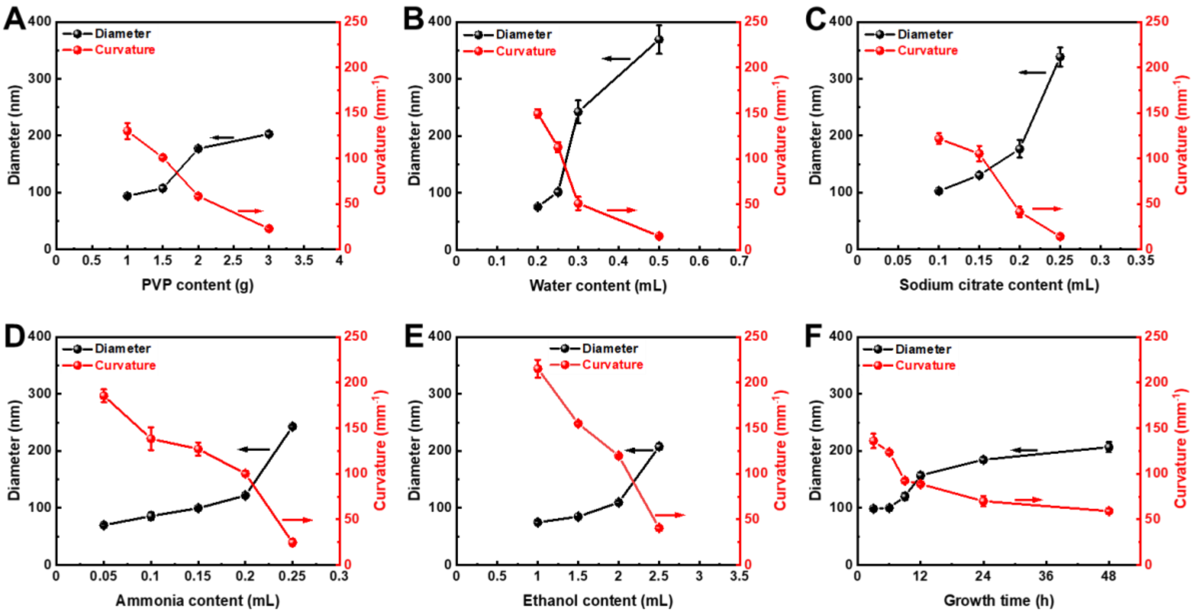

Figure S12. The summary of the diameter and curvature of as-prepared nanofibers and nanorods. (A) PVP content, (B) water content, (C) sodium citrate content, (D) ammonia content, (E) ethanol content, and (F) growth time.

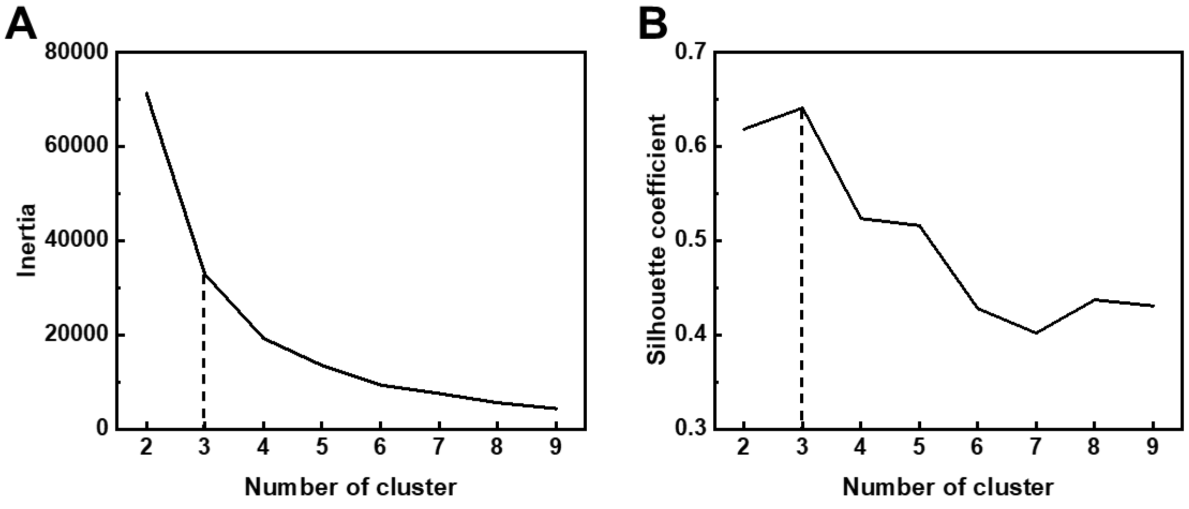

Figure S13. Two parameters to evaluate performance of clustering. (A) Inertia and (B) silhouette coefficient.

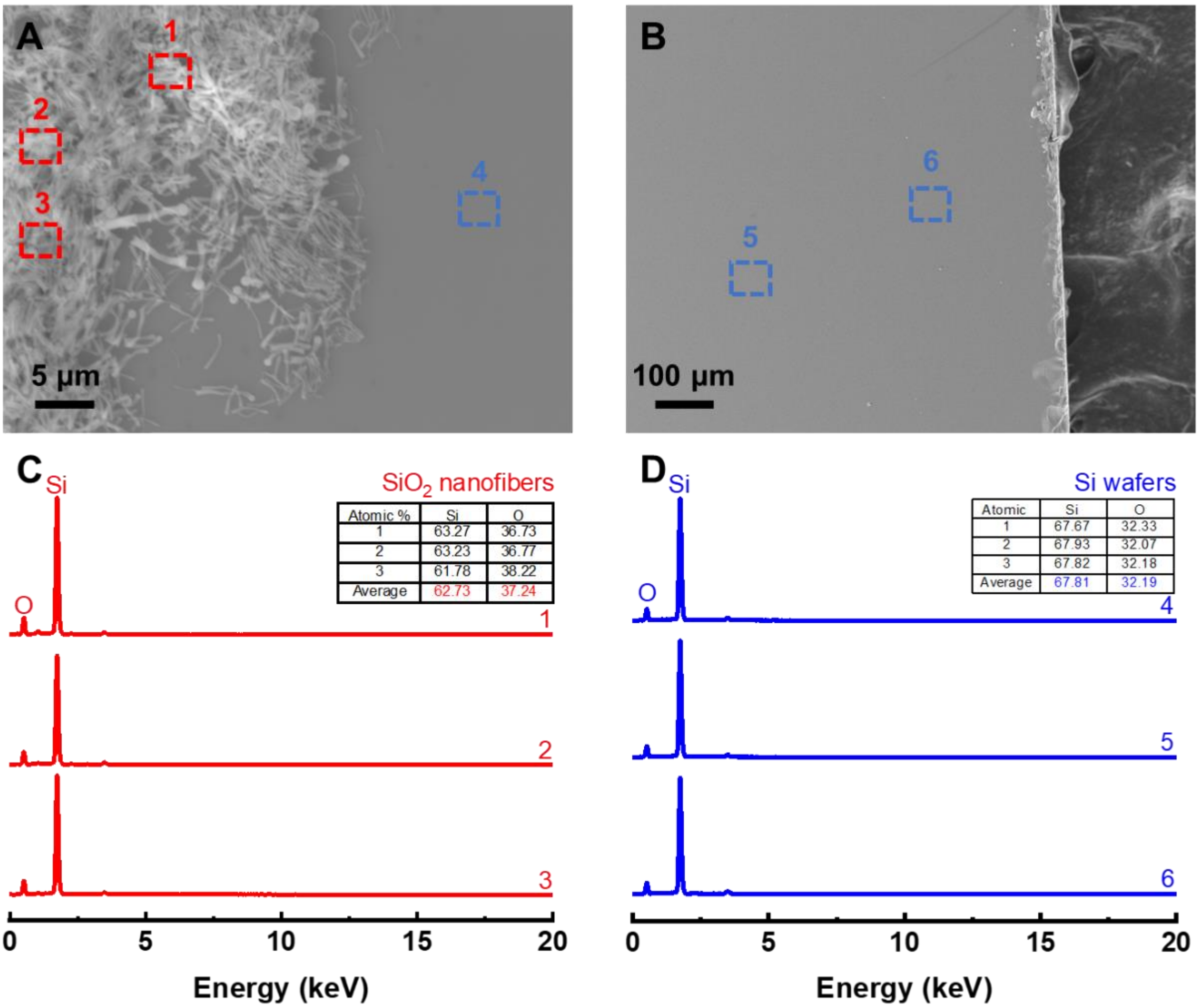

Figure S14. The similar Si and O elemental composition of silica nanofibers and the surface of silicon wafers. SEM images of (A) silica nanofibers and (B) silicon wafers. Energy-dispersive X-ray spectra of (C) silica nanofibers and (D) silicon wafers.

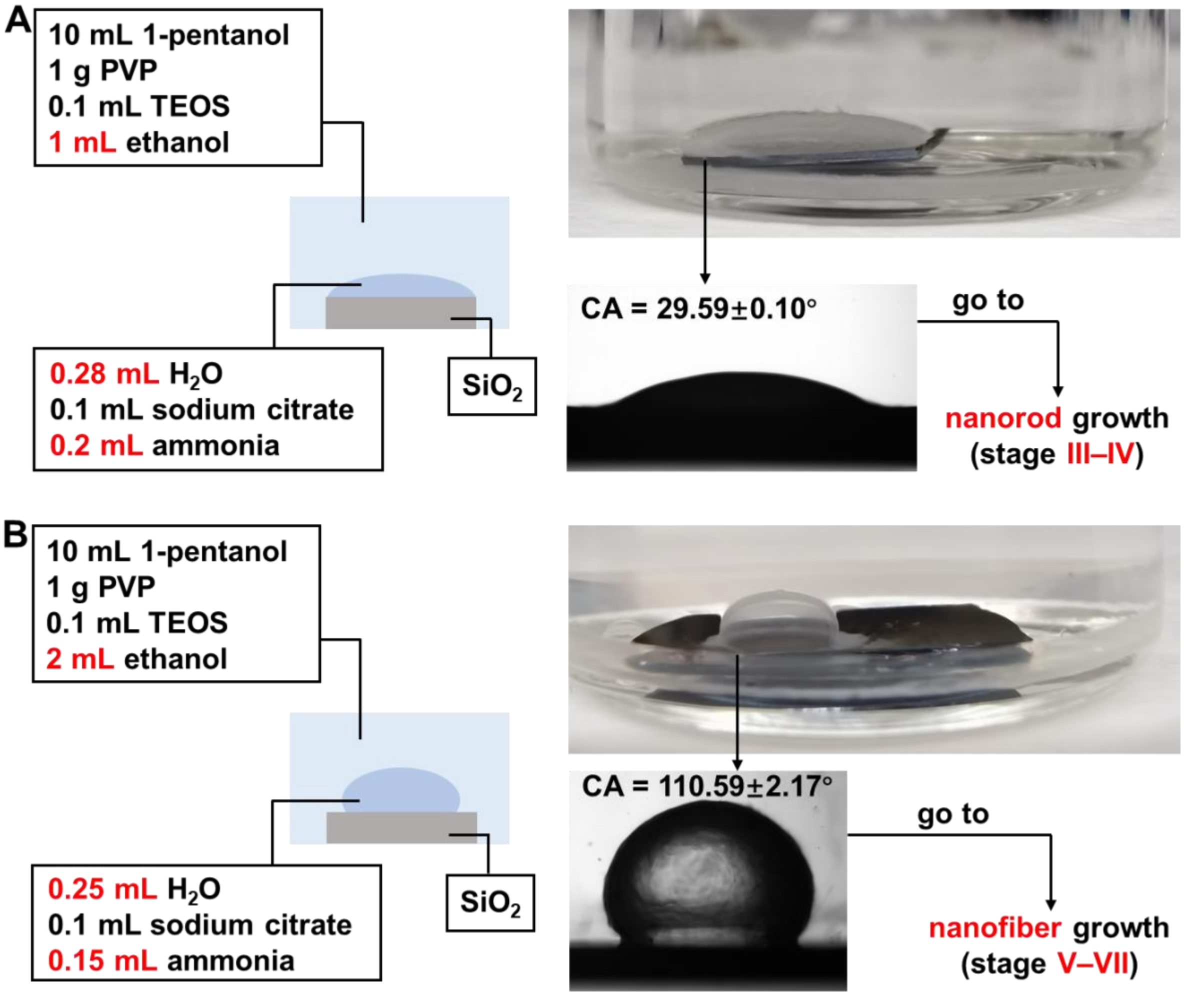

Figure S15. The macroscopic exploration of droplets on silica. The detailed composition, schematic illustrations, photographs, and contact angle measurements of (A) a system for imitating nanorod growth, and (B) a system for imitating nanofiber growth.

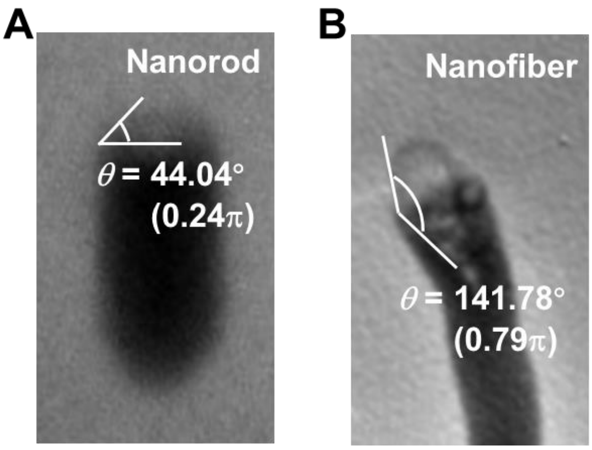

Figure S16. Contact angles of droplets on nanosilica. (A) Nanorod and (B) nanofiber measured from TEM images (Figure 2).

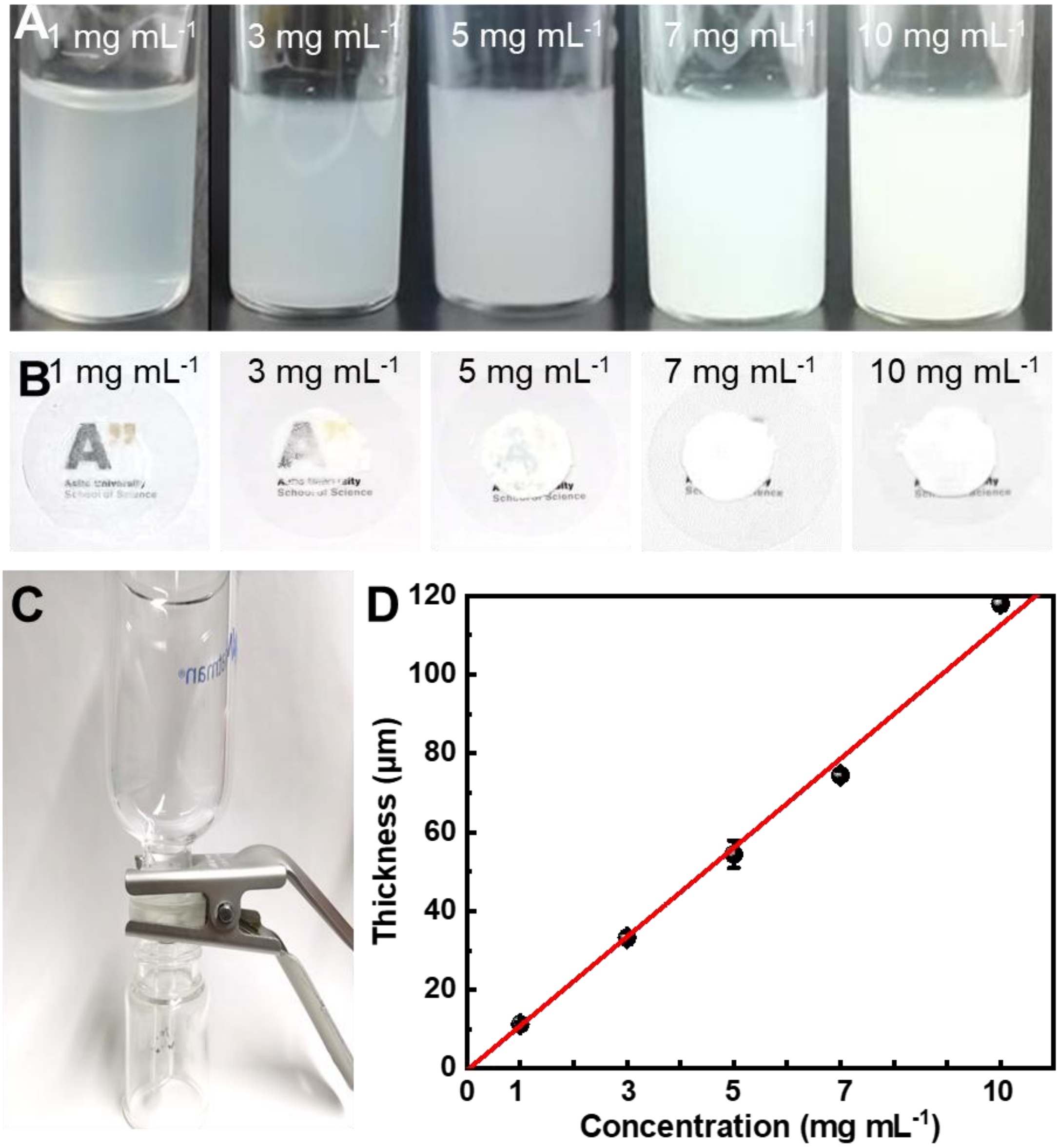

Figure S17. Formation of nanofiber films. (A) Photographs of nanofiber dispersions at different concentration (1, 3, 5, 7 and 10 mg mL^{-1}) in ethanol. (B) Photographs of nanofiber films prepared using (C) the instrument. (D) The relationship of the concentration of nanofiber dispersions and film thickness. Black dots are experimental data, and the red line is the linear fitting.

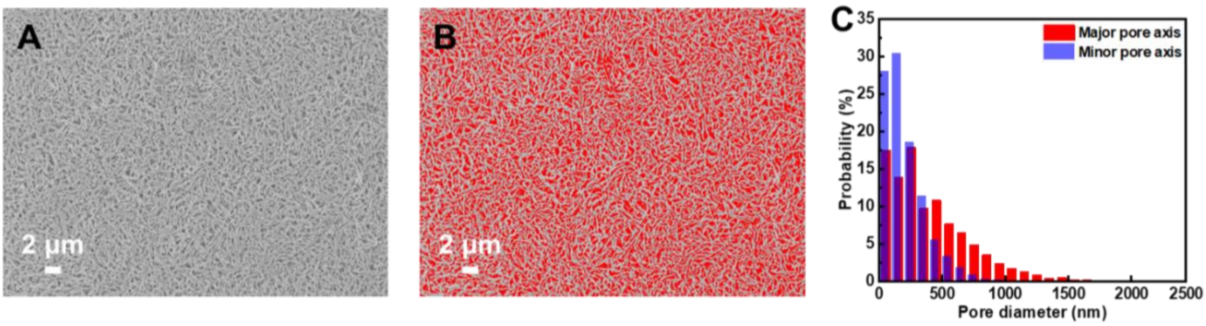

Figure S18. Characterization of nanofiber films. (A) SEM image of a nanofiber film and (B) its threshold image by ImageJ. (C) Pore size distribution of the film, including major and minor pore axes of ellipse pores.

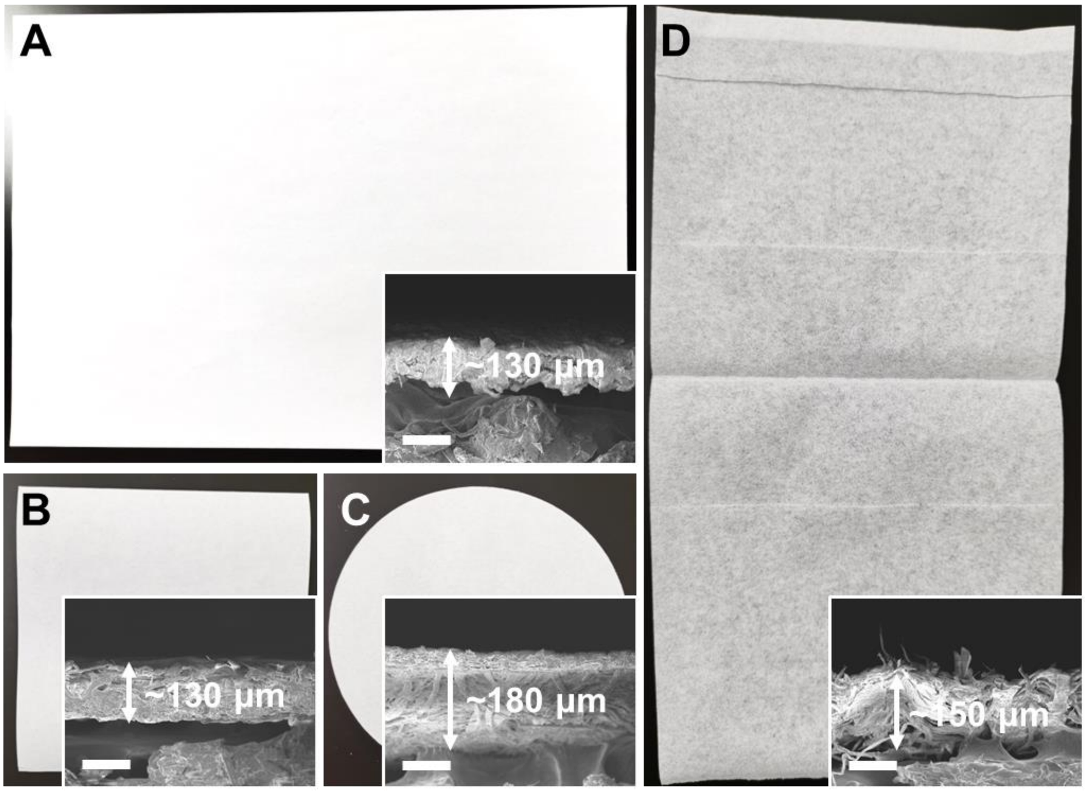

Figure S19. Characterization of commercial papers. Photographs of (A) copy paper (Papiers Clairefontaine A4-1844, 210×297 mm), (B) weighing paper (VWR 516-0318, 100×100 mm), (C) filter paper (Whatman 1454-090, diameter 90 mm) and (D) airlaid paper (Kimberly-Clark 05511, 110×210 mm), with inserted SEM images of the corresponding cross-sectional observation. The scale bars are 100 µm.

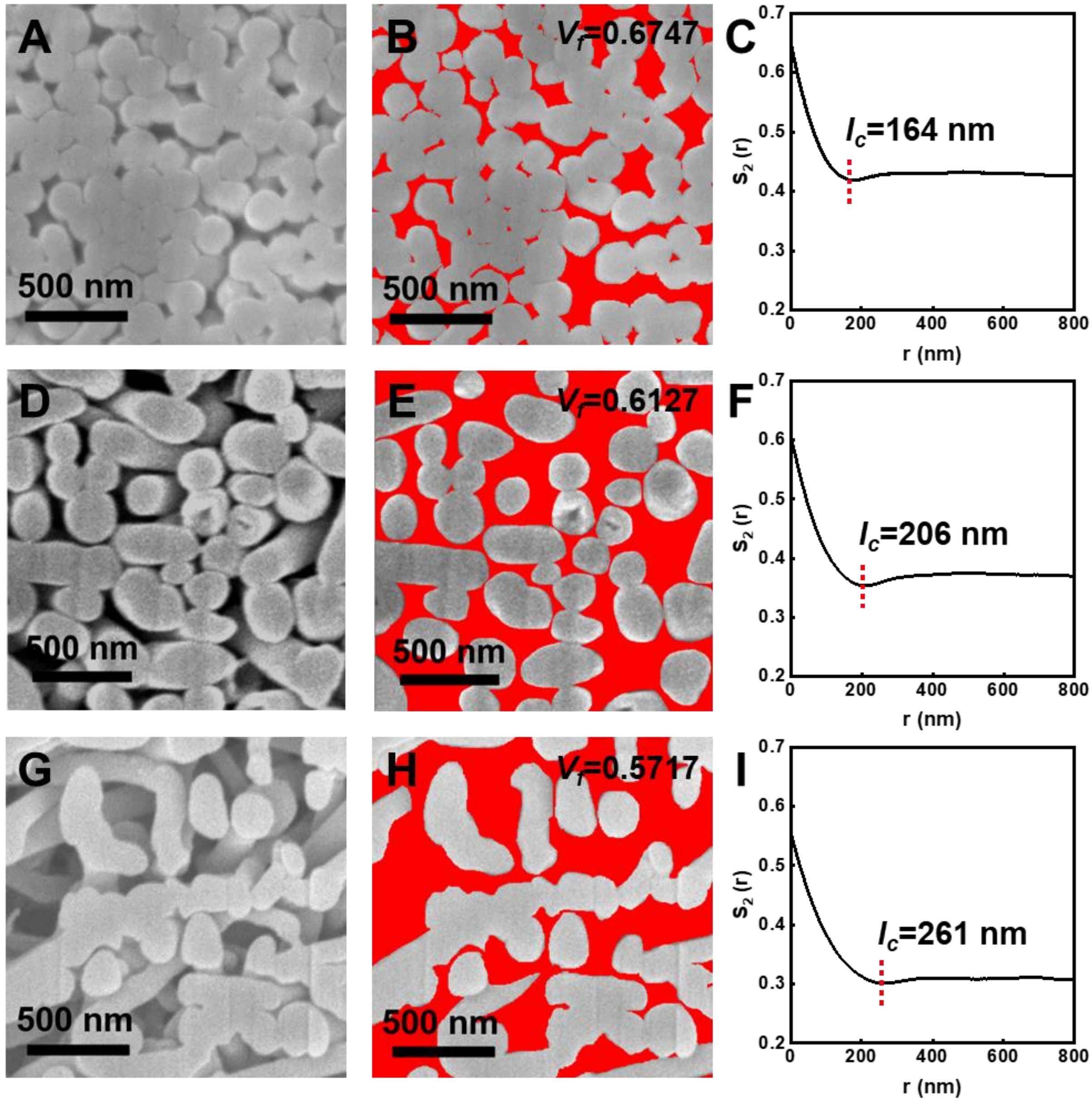

Figure S20. Filling fraction (V_f) and correlation length (l_c) measurements. (A) Milled cross-sectional image, (B) threshold image and (C) l_c function diagram of nanosphere film. (D) Milled cross-sectional image, (E) threshold image and (F) l_c function diagram of nanorod film. (G) Milled cross-sectional image, (H) threshold image and (I) l_c function diagram of nanofiber film.

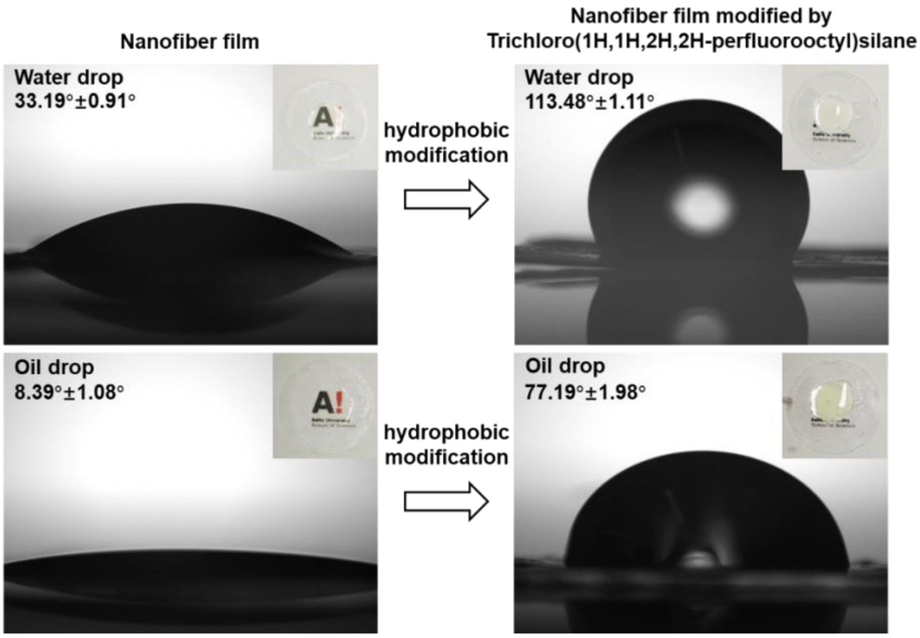

Figure S21. Modification of nanofiber film with a low-surface tension silane to preserve whiteness.

Supplemental Tables

Table S1. Parameters calculated from machine learning results.

Time	Inertia	Homo	Compl	V-means	ARI	AMI	Silhouette
0.015 s	32845	1.000	1.000	1.000	1.000	1.000	0.641

Table S2. Parameters of three light diffusion models calculated from experimental data fitting.

Model	Parameters			R-squared
Normal diffusion		A<br>0.02856		0.99480
Anomalous diffusion	A<br>0.03140		b<br>1.93924	0.99546
Photonic Ohm's law	a<br>0.00741	z_e<br>24.96840	z_p<br>20.73241	0.99961

Supplemental Notes

Note S1. Definition and calculation formulas of curvature, related to Figures 1C and S3.

In mathematics, the curvature is defined as the amount by which a curve deviates from being a straight line, which is usually used to characterize a curly degree at one point.[S1] Average curvature is an average value of curvature at multi-points along a curve, which represents the curly degree of the single curve. In this work, average curvature is introduced to characterize the curliness of silica nanofibers in different samples and implies an anisotropy degree. The schematic illustration of curvature measurements is shown in Figure S3. The calculation details of the curvature are shown as follows:

$$d\beta = \frac{y''}{1+y'^2}dx \qquad \text{(S1)}$$

$$ds = (1 + y'^2)^{0.5}dx \qquad \text{(S2)}$$

$$K = \lim_{\Delta s \to 0}\left|\frac{\Delta\beta}{\Delta s}\right| = \left|\frac{d\beta}{ds}\right| = \frac{|y''|}{(1+y'^2)^{1.5}} \qquad \text{(S3)}$$

$$r = \frac{1}{K} = \frac{(1+y'^2)^{1.5}}{|y''|} \qquad \text{(S4)}$$

where K is the curvature (mm^{-1}), $\Delta\beta$ is the tangent corner, Δs is the arc length between two neighboring points (M_1 and M_2), y' is the first derivative of the curve at the point when $\Delta s \to 0$ (M), y'' is the second derivative of the curve at the point when $\Delta s \to 0$ (M), r is curvature radius (nm).[S1,S2] Therefore, the curvature can be calculated as the reciprocal of the curvature radius, which can be measured by drawing osculating circles. Hereby, following the above principles, ImageJ with Kappa-Curvature Analysis module was used in our measurements facilitating the calculation.

Note S2. Equation derivation to show the influence of Brownian motion on silica nanofiber growth, related to Figures 3A–3C and Equations 1–3.

The diameter of nanofibers is determined by the basal areas of droplets. The curvature depends on the shape of nanofibers, which to some extent can be linked to the root mean square displacement (RMSD) of droplets within solvents during nanofiber growth. As the droplets are sufficiently small, they undergo continuous Brownian motion that impacts the RMSD of droplets within solvents.[S3,S4] Therefore, exploiting the correlation between the morphology of droplets and Brownian motion is essential to understand the nanofiber formation, further leading to better control over the morphology. First, the droplet on silica can be regarded as a spherical crown (Figure 3A).[S5] Herein,

$$h = R - R * \cos\theta, \tag{S5}$$

$$V = \pi R h^2 - \frac{\pi}{3}h^3 = \pi R^3(\frac{2}{3} - \cos\theta + \frac{1}{3}\cos^3\theta), \tag{S6}$$

where V is the volume of a spherical crown, h is the height of the crown, R is the radius of whole spheres, and θ is the contact angles of the crown, substrate, and ambience. As V is constant in Equation S6, both R and basal areas' diameter D can be expressed with the θ as follows:

$$R = \left(\frac{3V}{\pi}\right)^{\frac{1}{3}} * (2 - 3\cos\theta + \cos^3\theta)^{-\frac{1}{3}} = A * (2 - 3\cos\theta + \cos^3\theta)^{-\frac{1}{3}} \quad (A \text{ is constant}), \tag{S7}$$

$$D = 2 * R * \sin\theta = 2 * A * (2 - 3\cos\theta + \cos^3\theta)^{-\frac{1}{3}} * \sin\theta \quad (A \text{ is constant}), \tag{S8}$$

where A is defined as the volumetric constant. In addition, Brownian motion can be described by the Stokes–Einstein-Sutherland equation, that is, the RMSD $\langle x \rangle$ of droplets undergoing random walk in the solvent is both R- and θ-dependent:[S6-S9]

$$\langle x \rangle = \left(\frac{k_B T t}{3\pi\eta R}\right)^{\frac{1}{2}}, \tag{S9}$$

where k_B is the Boltzmann's constant, T is the absolute temperature, η is the dynamic viscosity, R is the radius of whole spheres, and t is the time.[S6-S9] Then, substituting Equation S7 into Equation S9 and setting T, η, V, and t as the constant, we get:

$$\langle x \rangle = \left(\frac{k_B T t}{3\pi\eta R}\right)^{\frac{1}{2}} = \left(\frac{{k_B}^3 * T^3 * t^3}{81 * \pi^2 * \eta^3 * V}\right)^{\frac{1}{6}} * (2 - 3\cos\theta + \cos^3\theta)^{\frac{1}{6}}$$

$$= B * (2 - 3\cos\theta + \cos^3\theta)^{\frac{1}{6}} \quad (B \text{ is constant}). \tag{S10}$$

Here, we just take the influence of Brownian motion on droplets into consideration. This is because the longitudinal size (1–5 μm) of nanofibers is significantly larger than the size of droplets (100–300 nm), as one of key factors that weaken the impact of the Brownian motions.

On the other hand, the density contrast ($\Delta\rho$) between silica solid (ρ_{silica}= 2.196 g cm^{-3}) and solvent ($\rho_{1\text{-}pentanol}$= 0.814 g cm^{-3}) is significantly larger than that between droplets (ρ_{water}= 1.000 g cm^{-3}) and solvent, resulting in sedimentation coefficient (s) of silica solid is also significantly larger than droplets due to:

$$s = \frac{\Delta\rho V}{\gamma}, \tag{S11}$$

where s is the sedimentation coefficient, $\Delta\rho$ is the density contrast between colloids and solvent, V is the colloidal volume, γ is the friction coefficient. That is, the "heavy" silica is more affected by gravity which weakens the influence of Brownian motion. Conversely, the "light" droplets are more susceptible to Brownian motion. Therefore, we consider that silica nanofibers remain "static", while droplets undergo Brownian motion for an easy model.

Afterwards, we can get that D is monotonically decreasing with θ within the range of (0, π) because of,

$$\frac{dD}{d\theta} = -\frac{2*A}{(2+\cos\theta)*(2-3\cos\theta+\cos^3\theta)^{\frac{1}{3}}}, \tag{S12}$$

where,

$$-1 < \cos\theta < 1, \tag{S13}$$

that is,

$$A > 0 \text{ and } 2+\cos\theta > 0 \text{ and } 2-3\cos\theta+\cos^3\theta > 0, \tag{S14}$$

Therefore,

$$\frac{dD}{d\theta} < 0. \tag{S15}$$

Similarly, $\langle x\rangle$ monotonically increases with θ within the range of (0, π) due to,

$$\frac{d\langle x\rangle}{d\theta} = \frac{B*\sin^3\theta}{2*(2-3\cos\theta+\cos^3\theta)^{\frac{5}{6}}}, \tag{S16}$$

where,

$$0 < \sin\theta < 1 \text{ and } -1 < \cos\theta < 1, \tag{S17}$$

that is,

$$B > 0 \text{ and } \sin^3\theta > 0 \text{ and } 2-3\cos\theta+\cos^3\theta > 0, \tag{S18}$$

Therefore,

$$\frac{d\langle x\rangle}{d\theta} > 0. \tag{S19}$$

Lastly, the RMSD $\langle x\rangle$ and diameter D is correlated, where $\langle x\rangle \propto (1/D)$,

$$\frac{d\langle x\rangle}{dD} = \frac{d\langle x\rangle}{d\theta} \times \frac{d\theta}{dD} < 0. \tag{S20}$$

The above mathematical derivation and simplification are supported and proved by Wolfram Mathematica (v. 13.1).

Note S3. Three light diffusion models, related to Figure 4G and Table S2.

Three light diffusion models including normal diffusion, anomalous diffusion, and photonic Ohm's law, were used for data fitting in Figure 4G.[S10-S12] The formulas are shown in the following, respectively:

$$T(L) = \frac{1}{1+AL}, \tag{S21}$$

$$T(L) = \frac{1}{1+AL^{b/2}}, \tag{S22}$$

$$T(L) = \frac{1}{az_e}\frac{\sinh[a(z_p+z_e)]\sinh(az_e)}{\sinh[a(L+2z_e)]}, \tag{S23}$$

where *T* is the transmittance and *L* is the thickness of the sample. *A* is a constant that depends on scattering mean free path, extrapolation length, and absorption. *b* is a parameter that describes anomalous diffusion behavior. *a* is the reciprocal of the absorption length (A smaller *a* indicates a larger absorption length and the less absorption of the film). z_e is the extrapolation length. z_p is the penetration length. The detailed parameters inferred from the fitting routine are shown in Table S2.

Supplemental References

S1. Mary, H., and Brouhard, G.J. (2019). Kappa (κ): analysis of curvature in biological image data using B-splines. Preprint at bioRxiv, https://doi.org/10.1101/852772.

S2. Ma, Y., Lan, K., Xu, B., Xu, L., Duan, L., Liu, M., Chen, L., Zhao, T., Zhang, J., Lv, Z., Elzatahry, A. A., Li, X., and Zhao, D. (2021). Streamlined mesoporous silica nanoparticles with tunable curvature from interfacial dynamic-migration strategy for nanomotors. Nano Lett. *21*, 6071–6079. https://doi.org/10.1021/acs.nanolett.1c01404.

S3. Goodarzi, F., and Zendehboudi, S. (2019). A comprehensive review on emulsions and emulsion stability in chemical and energy industries. Can. J. Chem. Eng. *97*, 281–309. https://doi.org/10.1002/cjce.23336.

S4. Langevin, D. (2022). Motion of small bubbles and drops in viscoelastic fluids. Curr. Opin. Colloid Interface Sci. *57*, 101529. https://doi.org/10.1016/j.cocis.2021.101529.

S5. Vafaei, S., and Podowski, M.Z. (2005). Analysis of the relationship between liquid droplet size and contact angle. Adv. Colloid Interface Sci. *113*, 133–146. https://doi.org/10.1016/j.cis.2005.03.001.

S6. Uhlenbeck, G.E., and Ornstein, L.S. (1930). On the theory of the Brownian motion. Phys. Rev. *36*, 823–841. https://doi.org/10.1103/PhysRev.36.823.

S7. Einstein, A. (1956). Investigations on the Theory of the Brownian Movement (Courier Corporation).

S8. Spiechowicz, J., Marchenko, I.G., Hänggi, P., and Łuczka, J. (2022). Diffusion coefficient of a Brownian particle in equilibrium and nonequilibrium: Einstein model and beyond. Entropy *25*, 42. https://doi.org/10.3390/e25010042.

S9. Bian, X., Kim, C., and Karniadakis, G.E. (2016). 111 years of Brownian motion. Soft Matter *12*, 6331–6346. https://doi.org/10.1039/C6SM01153E.

S10. Syurik, J., Jacucci, G., Onelli, O.D., Hölscher, H., and Vignolini, S. (2018). Bio-inspired highly scattering networks via polymer phase separation. Adv. Funct. Mater. *28*, 1706901. https://doi.org/10.1002/adfm.201706901.

S11. Toivonen, M.S., Onelli, O.D., Jacucci, G., Lovikka, V., Rojas, O.J., Ikkala, O., and Vignolini, S. (2018). Anomalous-diffusion-assisted brightness in white cellulose nanofibril membranes. Adv. Mater. *30*, 1704050. https://doi.org/10.1002/adma.201704050.

S12. Caixeiro, S., Peruzzo, M., Onelli, O.D., Vignolini, S., and Sapienza, R. (2017). Disordered cellulose-based nanostructures for enhanced light scattering. ACS Appl. Mater. Interfaces *9*, 7885–7890. https://doi.org/10.1021/acsami.6b15986.
